# Evaluating molecular docking for binding affinity predictions: a systematic analysis of key parameters and the utility of AlphaFold2 structures for the Schrödinger dataset

**DOI:** 10.1007/s10822-026-00929-9

**Published:** 2026-08-29

**Authors:** Konstantinos Tornesakis, Jonathan W. Essex, Paul A. Cox, Gerhard König

**Affiliations:** 1 Centre for Enzyme Innovation, University of Portsmouth, St. Michael’s Building, Portsmouth, PO1 2DT UK; 2 School of Chemistry, University of Southampton, Highfield, Southampton, SO17 1BJ UK; 3 School of Medicine, Pharmacy and Biomedical Sciences, University of Portsmouth, St. Michael’s Building, Portsmouth, PO1 2DT UK

**Keywords:** Molecular docking, Binding affinity prediction, AlphaFold2, Scoring functions

## Abstract

Molecular docking is one of the most established methods in computational drug discovery, due to its balance of speed and accuracy. However, the accuracy of docking results depends on a number of different parameters, and systematic reference data for comparisons to more advanced methods for binding affinity prediction are still scarce. This study assesses the impact of key parameters on the accuracy of binding free energy estimates from docking, using nine benchmark systems with 278 high-affinity ligands. Using the Molecular Operating Environment (MOE), we evaluated combinations of three receptor structures (two crystal structures, one AlphaFold2 model), two force fields, two scoring functions, two receptor flexibility settings, and two statistical evaluation schemes. The performance of the docking approaches is measured based on the squared Pearson’s correlation coefficient (R²), the root mean square error (RMSE) with respect to the experimental binding affinities, as well as the mean signed error (MSE) and Kendall’s tau for individual targets and the full dataset. The results show that the scoring function and the protein structure are the most important factors for binding affinity accuracy in rigid docking with the MOE software. Amber10:EHT and MMFF94x force fields had the same average R_mean_^2^ value, but Amber10:EHT had a lower average RMSE_mean_. AlphaFold2 protein models yielded lower binding affinity accuracy and higher errors compared to experimental crystal structures, although induced fit docking improved results. Using the original benchmark, we also compared several docking programs. DOCK6 and MOE performed best, with mean R² values of about 0.49 and 0.40, respectively. The remaining docking programs did not outperform a molecular weight regression baseline. For a subset of four targets (CDK2, JNK1, P38, TYK2) evaluated in previous work, the performance of the optimized DOCK6 and MOE protocols produced correlation coefficients similar to those reported for certain MM/PBSA, FMO, and Boltz2 implementations evaluated on the same target subset. This raises questions about potential dataset biases, the structural preparation, or the implementation of those methods. Docking therefore should be considered as an important and computationally inexpensive reference baseline for binding affinity prediction.

**Supplementary Information:**

The online version contains supplementary material available at https://doi.org/10.1007/s10822-026-00929-9.

## Introduction

Modern drug design relies upon in-silico approaches to calculate target and ligand properties. The calculation of binding affinities of ligands toward their target is central for potency and selectivity [1]. However, depending on the method, significant amounts of computational time are required to predict protein-ligand binding. This is especially the case during lead optimization, where several ligands may need to be tested [2]. This challenge highlights the need for reliable initial docking predictions, as the efficiency of downstream calculations depends on the quality of the starting poses. Recent work by Sindt, Bret and Rognan highlights that the efficiency of ultralarge virtual screening campaigns is also primarily determined by the accuracy of the binding affinity predictions from the docking procedure itself [3]. Further refinement with enhanced rescoring methods such as quantum-mechanical calculations, molecular mechanics with implicit solvent, or machine learning, provided only marginal improvements, due to their dependency on the docking pose [3]. This surprising finding raises the question whether more advanced methods are really significantly better than docking when it comes to predicting protein-ligand binding affinities.

A wide range of computational strategies exist for predicting protein-ligand binding, including docking, free energy simulations, MM-GBSA, quantum-mechanical methods, and machine learning models [4–7]. Because of the different levels of theoretical rigor, it is expected that the different binding affinity prediction methods exhibit different levels of binding affinity accuracy [8]. Therefore, the objective evaluation of their performance is crucial, in order to inform researchers about their strengths and weaknesses. Several benchmarking studies have been reported for free energy simulations [2, 9–10]. Important aspects of the benchmark systems include the use of well-characterized drug targets with a series of high-quality protein structures, and a sufficiently large number of ligands that cover both a diverse chemical space and a large dynamic range of binding affinities [9]. Among the widely used benchmark systems for free energy simulations are those developed by Schrödinger, Merck, and a collaboration between Janssen, Boehringer-Ingelheim, and the Max Planck Institute for Biophysical Chemistry [1, 11–13]. The JACS Schrödinger dataset consists of eight well-characterized drug targets (BACE1, Thrombin, PTP1B, MCL-1, Tyk2, JNK1, P38 and CDK2) and 199, known, high-affinity ligands [14]. For this dataset, several free-energy simulation studies have been performed, evaluating Amber/TI calculations, Flare’s FEP and Gromacs simulations, as well as machine learning protocols [15–17]. While Glide scores and MM/GBSA results were reported in the original paper, no other docking score results have been evaluated for this set, which would be an important baseline for evaluating the binding affinity prediction accuracy of other models.

In this study, we address this gap by evaluating the accuracy of binding free energy estimates from Molecular Operating Environment (MOE) scoring functions in molecular docking. The well-established MOE platform was selected because it directly predicts binding free energies as part of its molecular docking engine [18]. To enable a comprehensive assessment, we constructed an expanded version of the JACS Schrödinger dataset, termed JACS Schrödinger*+*. This version included all targets from the work of Wang et al. (BACE1, Thrombin, JNK1, MCL-1, CDK2, PTP1B, P38 and Tyk2), as well as the c-MET system from the Merck dataset [1, 14]. Furthermore, additional experimental data were incorporated for the ligand datasets of BACE1, Thrombin, JNK1, MCL-1, PTP1B and P38 targets [19–28]. The number of ligands in the original experimental publications that were used to collect the data for the Schrödinger benchmark set in Reference 14 was higher than in the final benchmark set and several ligands were omitted without specific justification. The missing ligands were included to avoid potential selection bias from the ligand selection in the Schrödinger data set [14]. Our approach was inspired by Goel et al., who utilized an extended ligand dataset for the protein targets of the same benchmark [29].

The goal of this work is to establish how well molecular docking can reproduce binding affinities relative to more computationally demanding methods while maintaining high efficiency. We further evaluate how key methodological factors, such as scoring function, force field, receptor structure source (including AlphaFold2 models), receptor flexibility and presence of water molecules influence docking performance in the MOE platform. These analyses aim to provide a fair and transparent baseline for evaluating docking binding affinity accuracy and to inform future developments in computational binding-affinity prediction.

## Methods

### Selected datasets

The benchmark set of this report (JACS Schrödinger+) was derived from the JACS Schrödinger dataset and expanded with additional experimental data to improve chemical and structural diversity [19–28]. It includes nine protein targets, BACE1, Thrombin, PTP1B, MCL-1, Tyk2, JNK1, P38, CDK2 and c-MET, comprising a total of 278 protein-ligand complexes (Table 1). The c-MET system was included from the Merck dataset [1, 9, 14]. For several targets (BACE1, Thrombin, JNK1, MCL-1, PTP1B and P38), missing ligands reported in the primary literature were reintroduced to ensure completeness and avoid selection bias [19–28].

**Table 1:** Target proteins and their structures for the JACS Schrödinger+ dataset. The “+” indicates protein targets with additional experimental data relative to those in the original JACS Schrödinger benchmark

Protein target	Crystal structures (Resolution Å, number of residues) + AlphaFold2 (number of residues)	Number of ligands
BACE1+	4DJW (1.90, 390), 4DJY (1.86, 390), AF2 (390)	43
Thrombin+	2ZDA (1.73, 258), 5JZY (1.27, 257), AF2 (258)	12
c-MET	4R1Y (2.00, 286), 5EOB (1.75, 292), AF2 (292)	24
JNK1+	2GMX (3.50, 357), 3ELJ (1.80, 358), AF2 (369)	59
MCL-1+	4HW3 (2.40, 149), 4WMU (1.55, 521), AF2 (522)	47
CDK2	1H1Q (2.50, 297), 3DDQ (1.80, 298), AF2 (303)	16
PTP1B+	2QBS (2.10, 297), 2HB1 (2.00, 297), AF2 (299)	26
P38+	3FLY (1.80, 349), 3FLN (1.90, 350), AF2 (355)	35
Tyk2	4GIH (2.00, 288), 3LXP (1.65, 291), AF2 (296)	16

### Protein and ligand preparation

Experimental crystal structures were downloaded from the Protein Data Bank, while corresponding AlphaFold2 models were generated using the ColabFold implementation [30–32]. Multiple sequence alignments were generated using the default MMseqs2 search implemented in ColabFold against the default ColabFold databases, followed by structure prediction with the AlphaFold2_ptm model. No template was used for the generation of the models, the number of recycles was set to 3 and the maximum number of relaxation iterations to 200. Only a single protein chain was retained for each target. All non-essential water molecules were removed except those mediating key ligand interactions, which were identified from crystallographic complexes and introduced into AlphaFold2 models after structural alignment.

Protein structures were protonated with MOE’s Protonate3D function and missing residues or loops were reconstructed using the Structure Preparation and Loop Modeler tools. Ligands were prepared within MOE and energy-minimized under each tested force field. Conformational sampling during docking was limited to rotatable bonds, whereas cyclic substructures retained the conformations present following ligand preparation. For BACE1, alternative receptor protonation and ligand tautomeric states were evaluated to account for the possible mono-protonation of catalytic aspartates and tautomeric ligand forms.

### Molecular docking protocol

The Dock function in MOE was used to dock the ligand datasets to the receptors. The binding site was defined using the Selected Residues option, including all residues forming direct or secondary interactions with ligands in experimentally determined crystal structures. Ligand placements were generated using the Triangle Matcher algorithm, sampling up to 1000 poses per ligand for rigid docking.

MOE docking consists of three primary stages, pose generation, scoring and refinement with rescoring. For pose generation, ligand conformers are created by rotating torsion angles and placed within the active site using geometric matching algorithms. With respect to scoring, each pose is evaluated using empirical or physics-based scoring functions to approximate binding free energy, considering electrostatic, van der Waals, hydrophobic and other interactions. Finally, the top poses are minimized using the selected force field, for refinement, and rescored for ranking.

For induced-fit docking, receptor side-chain atoms within 6 Å of the ligand were allowed to move (Free option), while backbone atoms were kept fixed. Selected side chains were fully flexible in their dihedral angles during docking to relieve steric clashes and optimize local protein-ligand complementarity. The number of adjusted residues therefore varied depending on ligand size and binding mode, and was determined dynamically by the spatial cutoff criteria (BACE1 20–24, CDK2 14–18, JNK1 15–17, MCL-1 13–17, P38 15–19, PTP1B 16–24, thrombin 17–19, Tyk2 16–18). To balance computational cost, 250 poses per ligand were generated for flexible docking.

Two scoring functions were evaluated, GBVI WSA dG (GBVI) and Affinity dG. GBVI WSA dG is a force field-based solvation model that estimates the binding free energy from Coulombic, van der Waals, and solvation contributions [18, 33]. Affinity is a semiempirical linear model combining hydrogen bonding, ionic, hydrophobic, and metal-ligation terms [18, 34]. The two scoring functions were selected because Affinity dG outperformed all other scoring functions in MOE in a previous study [34], while GBVI is the new default scoring function in MOE, and was not part of the previous comparison.

Two force fields were tested, MMFF94x and Amber10:EHT. MMFF94x is optimized for small organic molecules, and Amber10:EHT is parameterized for proteins and small molecules [35–36]. Binding affinities were computed using either the top-ranked docking pose or the average of the five best poses to account for conformational entropy [37].

### Performance evaluation and computational setup

Docking binding affinity accuracy was assessed by comparing predicted binding free energies with experimental affinities (converted to ΔG values). Statistical performance metrics included the squared Pearson’s coefficient (R²), root mean square error (RMSE), mean signed error (MSE), and Kendall’s Tau. The minimum, maximum and range of experimental ΔG values for each target dataset are reported in Supplementary Table 1. Pose quality was evaluated based on the heavy-atom root-mean-square deviation (RMSD) between docked and crystallographic ligand coordinates, computed using CalcLigRMSD [38–39]. The pose reproduction accuracy of predicted ligand poses was evaluated only for ligands whose experimentally determined binding poses were available from protein-ligand complex structures.

To systematically assess the influence of the investigated parameters, a two-step analysis strategy was employed. A fully combinatorial evaluation of all parameter permutations across all protein targets would result in a prohibitively large number of docking calculations (9 targets × 3 structures × 2 scoring functions × 2 force fields × 2 receptor flexibility settings × 2 analysis schemes = 432 permutations). Therefore, all combinations of scoring function, force field, receptor structure, receptor flexibility, and analysis scheme were evaluated for two representative systems (Thrombin + and c-MET), which are well-established benchmark targets with different binding site characteristics. This exhaustive subset analysis with 96 permutations was used to verify that the relative trends in performance are robust with respect to the choice of parameters. Based on these results, a consistent protocol was applied to the full dataset of nine protein targets, using both scoring functions, all available receptor structures, rigid docking, and the selected force field and analysis scheme, to evaluate overall trends across all targets. Afterwards, the effect of receptor flexibility was evaluated with this optimized setup. This approach allows identification of the most relevant factors while maintaining computational feasibility.

All calculations were performed on an 8-core workstation running MOE 2022.03 under Linux. The docking protocols were executed using the same random seed to ensure reproducibility. Default convergence criteria were applied unless otherwise noted. Further details of the docking setup, scoring equations and force field parameters are provided in the Supplementary Information.

### Evaluations of alternative docking tools

Flexible docking with Smina, AutoDock Vina, and GNINA were run with the same receptor and ligand inputs, the same docking box, exhaustiveness 8, nine output poses per ligand, and the same per-ligand random seed [40–42]. Smina used the default Vina-compatible scoring function, not Vinardo. Receptor side-chain flexibility was enabled through the flex receptor definition, while the ligand remained fully flexible inside the box. The top-ranked pose, mode 1, was used as the primary prediction. For GNINA, the Vina-style search was combined with CNN-based rescoring. We recorded mode 1 Vina score, CNNaffinity, and CNNpose.

DOCK6 was run in flexible ligand mode (anchor-and-grow with minimization) [43]. Receptors were converted to Sybyl-typed MOL2 with Amber ff14SB partial charges via pdb4amber and tleap. Crystallographic waters were omitted from DOCK6 receptors. Ligands were assigned atom types and AM1-BCC partial charges with antechamber. Coordinates matched those used for the AutoDock prep (Schrödinger reference geometry, or MOE-transferred chemistry where applicable). Binding-site spheres were generated from reference-ligand heavy-atom coordinates, reduced to at most 200 spheres with 1.5 Å radius using a fixed random seed of 42, and used to build target-specific energy grids. The grid enclosure was derived from the sphere set with an additional 10 Å margin on all sides. Importantly, DOCK6 “flex” denotes anchor-and-grow ligand conformational search against a rigid receptor; it does not implement the receptor side-chain flexibility used by Smina, Vina, and GNINA. For each ligand, automated matching and orientation were enabled (maximum 50 orientations, minimum anchor size of five heavy atoms, interatomic distance tolerance 0.25 Å). Conformer pruning used up to 100 orientations per cluster, with orienting and conformer score cutoffs of 1000 and 25, respectively; internal ligand strain was included (repulsive exponent 12, cutoff 100). Scoring used the primary grid score with van der Waals, electrostatic, and repulsive scaling factors of 1.0. Minimization was applied to the anchor, growing fragments, and full ligand using a single simplex cycle (score convergence 0.1, cycle convergence 1.0, up to 500 iterations for anchor and growth steps). One final pose per ligand was retained and ranked; the grid score of the top-ranked pose was used for correlation analysis. Grid scores are reported in DOCK6 energy units and were not treated as absolute kcal/mol values.

## Results

### Effect of scoring function

The first aim was to evaluate the relative performance of two MOE scoring functions. GBVI WSA dG is MOE’s default scoring function, while the Affinity dG scoring function previously showed the highest success rates for crystal structures among all other scoring functions in MOE [18, 33–34].

**Fig. 1:**
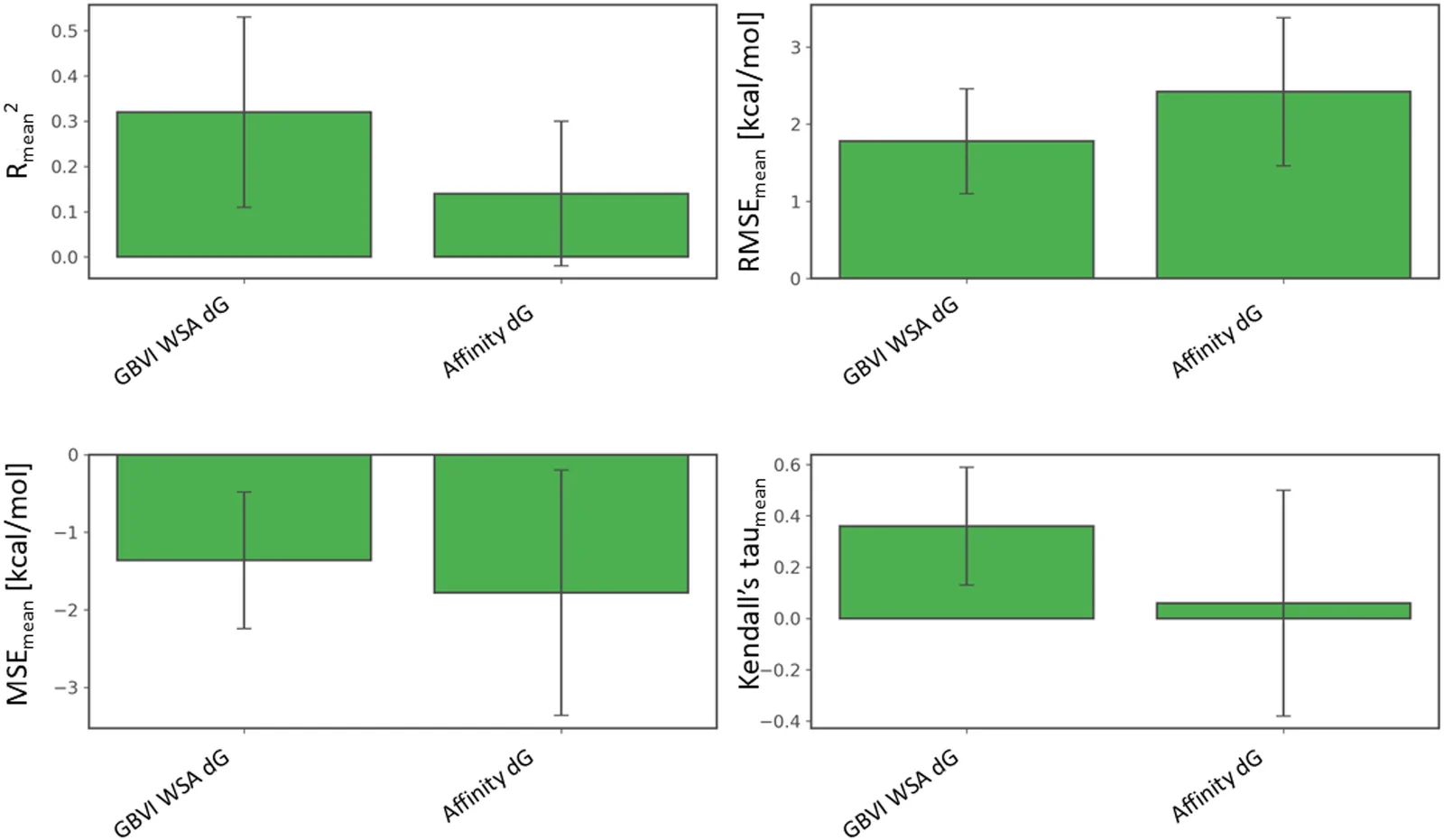
Comparison of metrics with the GBVI and Affinity dG scoring functions for the JACS Schrödinger+ dataset. The mean values and standard deviations of R^2^, RMSE, MSE and Kendall’s Tau of the binding scores are calculated over all protein targets and all protein structures using rigid docking with the Amber10:EHT force field

The R_mean_^2^, RMSE_mean_, MSE_mean_ and Kendall’s Tau_mean_ for the GBVI and Affinity scoring functions across all targets, calculated as averages of all protein structures, using rigid docking and the Amber10:EHT force field, are presented in Fig. 1. The results for the individual targets can be found in Supplementary Tables 2 and 3. Amber10:EHT is the default force field in MOE, and the impact of force field selection on docking performance will be evaluated in the next section. Figure 1 shows that the GBVI scoring function performs better than the Affinity scoring function in terms of R_mean_^2^ (0.32 versus 0.14), RMSE_mean_ (1.78 versus 2.42 kcal/mol) and MSE_mean_ values (-1.36 versus − 1.78 kcal/mol). Further analysis, using the Kolmogorov-Smirnov test, validated that the differences in R_mean_^2^, RMSE_mean_ and Kendall’s Tau_mean_ metrics were statistically significant. However, the performance of the scoring function strongly depends on the target and the employed protein structure, leading to high error bars for the average metrics. Based on this evaluation, the GBVI scoring function was used throughout the remainder of the manuscript.

To ensure that the use of the Amber10:EHT force field did not bias the results, additional calculations with the MMFF94x force field were performed on the Thrombin + and c-MET benchmarks [1, 44]. The R_mean_^2^, RMSE_mean_, MSE_mean_ and Kendall’s Tau_mean_ for the GBVI and Affinity scoring functions calculated as averages over all targets and structures, based on the two force field options and using the top docking pose from rigid docking, are presented in Supplementary Tables 4, 5, 6 and 7. For the evaluated permutations over this subset, the GBVI scoring function also outperformed the Affinity dG with a higher R_mean_^2^ (0.41 versus 0.16), lower RMSE_mean_ (1.33 versus 1.89 kcal/mol) and lower MSE_mean_ (-0.73 versus − 1.35 kcal/mol). The R^2^ and Kendall’s Tau in c-MET, the RMSE in Thrombin and the R_mean_^2^, MSE_mean_ and Kendall’s Tau_mean_ across both targets showed statistically significant differences between the two scoring functions. The exhaustive analysis on the Thrombin + and c-MET systems therefore verifies that the relative performance of the scoring functions is consistent across different combinations of parameters. The agreement between these results and the analysis over the full dataset supports the use of GBVI as a robust choice for subsequent evaluations.

### Effect of force field

Force fields can influence docking outcomes by affecting refinement and scoring functions such as GBVI WSA dG. Here, the Amber10:EHT and MMFF94x force fields in MOE were evaluated, both designed for small molecules. MMFF94x is based on quantum-mechanical calculations, and Amber10:EHT employed the extended Hückel method [35–36].

**Fig. 2:**
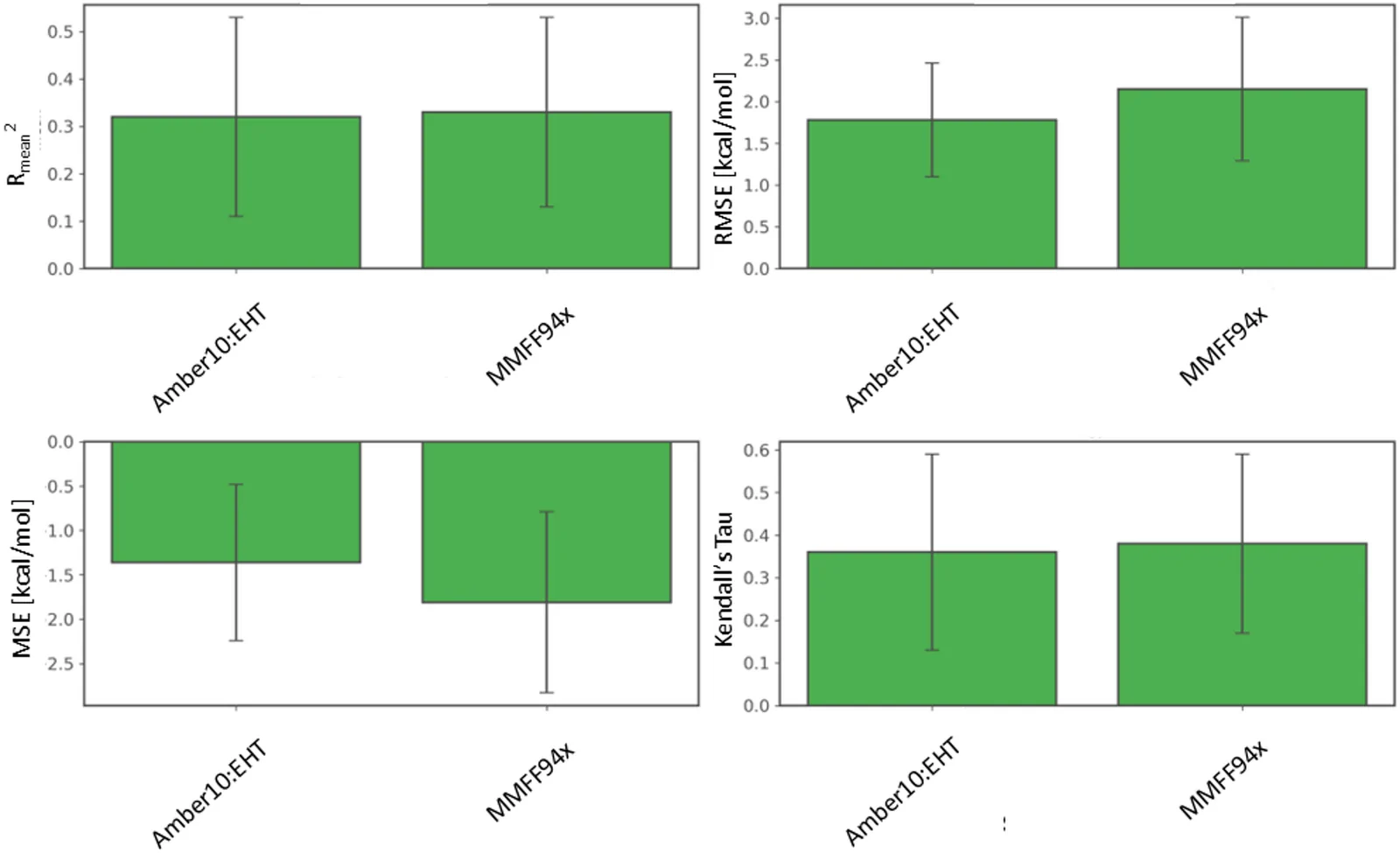
Comparison of metrics with the MMFF94x and Amber10:EHT force fields for the JACS Schrödinger+ dataset. The mean values and standard deviations of R^2^, RMSE, MSE and Kendall’s Tau of the binding scores are calculated over all protein targets and all protein structures using rigid docking with the GBVI scoring function

Performance was assessed across all nine benchmark systems of the JACS Schrödinger+ dataset using rigid docking with the GBVI scoring function. The resulting values of R_mean_^2^, RMSE_mean_, MSE_mean_ and Kendall’s Tau_mean_, calculated as an average over all targets and structures, based on rigid docking with the GBVI scoring function and the top binding affinity pose are presented in Fig. 2. The results for the individual targets can be found in Supplementary Fig. 1 and Supplementary Tables 8 and 9. Both force fields showed about the same level of correlation (R_mean_^2^ of 0.32 for Amber10:EHT versus 0.33 for MMFF94x). However, the Amber10:EHT yielded a lower average RMSE_mean_ than MMFF94x  (1.78 kcal/mol versus 2.15 kcal/mol). Further analysis, using the Kolmogorov-Smirnov test, did not detect a statistically significant difference at the selected significance threshold. However, the absence of statistical significance should not be interpreted as evidence of equivalence, particularly given the small number of target systems and the large variations across targets. The Amber10:EHT force field was used for subsequent calculations, due to the lower RMSE_mean_ value compared to MMFF94x.

### Consideration of multiple binding poses

Docking based affinity estimates are commonly derived from the score of the highest ranked pose. Previous work has suggested that incorporating information from multiple poses may improve agreement with experimental affinities, particularly when several plausible binding modes are sampled [37]. We therefore compared binding affinity estimates obtained from the highest ranked pose, TOP1, with the arithmetic mean of the five highest ranked poses, TOP5.

**Fig. 3:**
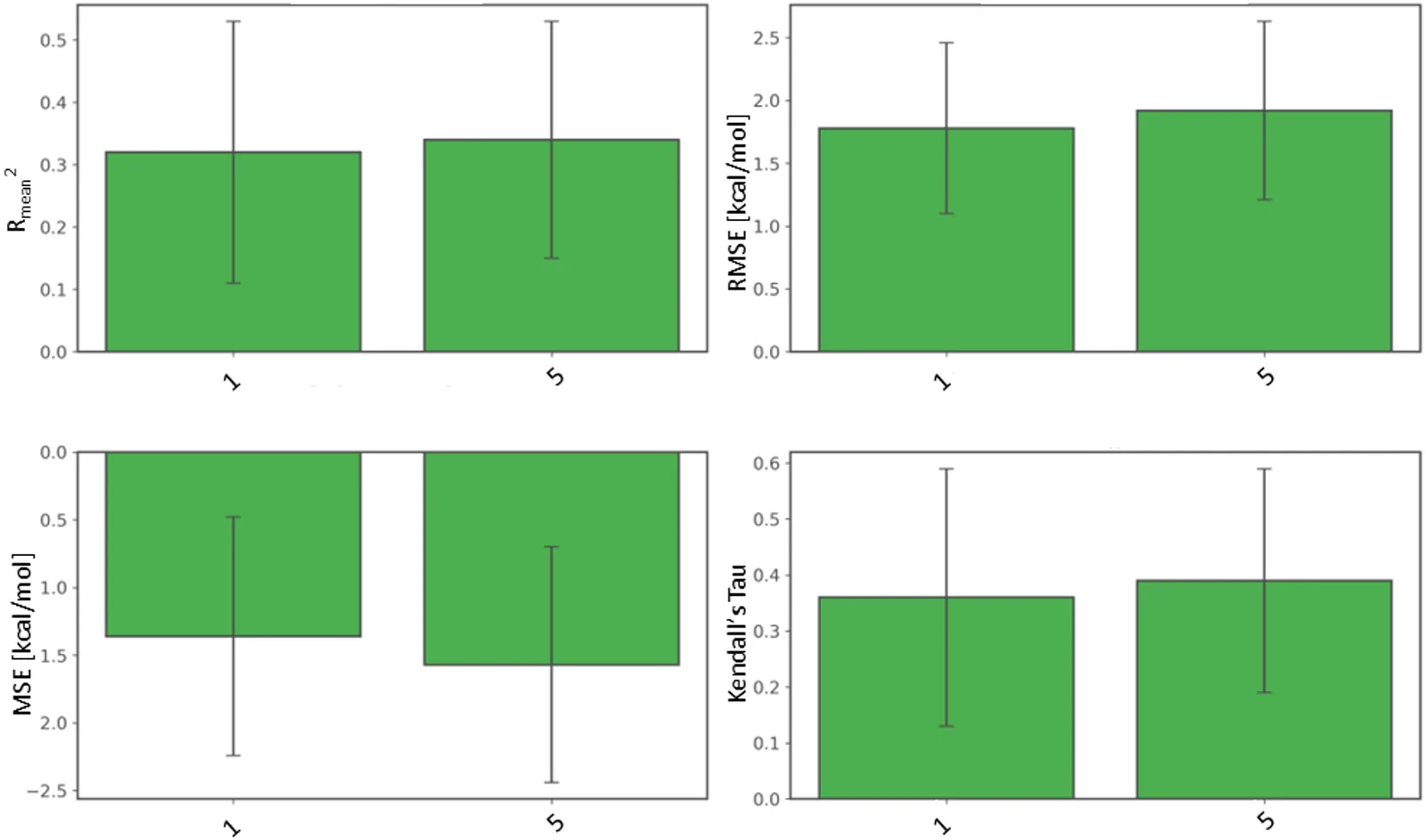
Comparison of employing the highest affinity binding pose (TOP1) and averages of the top five highest affinity binding poses (TOP5), for the JACS Schrödinger+ dataset. The mean values and standard deviations of R^2^, RMSE, MSE and Kendall’s Tau of the binding scores are reported

As shown in Fig. 3, TOP1 and TOP5 produced similar affinity ranking performance. The mean R^2^ increased slightly from 0.32 for TOP1 to 0.34 for TOP5, while the corresponding mean Kendall’s tau values were 0.36 and 0.39, respectively. However, TOP1 produced lower absolute errors, with a mean RMSE of 1.78 kcal/mol compared with 1.92 kcal/mol for TOP5. The corresponding mean signed errors were − 1.36 and − 1.57 kcal/mol.

As an additional analysis, we calculated a Boltzmann-weighted mean of the five highest ranked poses, denoted Boltzmann5. This approach produced results intermediate between TOP1 and TOP5, with a mean R^2^ of 0.33, Kendall’s tau of 0.38, RMSE of 1.89 kcal/mol, and mean signed error of −1.51 kcal/mol. The complete Boltzmann5 results are reported in Supplementary Table 15.

Neither arithmetic nor Boltzmann weighted averaging produced a consistent improvement over the highest ranked pose. Kolmogorov Smirnov testing did not detect statistically significant differences among the three approaches at the selected significance threshold. TOP1 was therefore retained for subsequent analyses because it yielded the lowest RMSE and the smallest mean signed error and required no additional pose aggregation.

### Effect of using AlphaFold2 structures

The binding affinity prediction accuracy of docking predictions depends on the quality of the protein structure used. For many targets, numerous crystal structures are available in the RCSB protein data bank (PDB) [31]. However not all deposited structures are suitable for docking due to missing domains close to the binding pocket or artefacts due to the crystallization conditions. Another important factor is the presence of crystal waters, as many protein-ligand interactions rely on bridging water molecules and their absence can lead to underestimated binding affinities. Furthermore, the orientation of binding site residues might differ significantly in the holo and apo form and may vary depending on the bound ligand.

To test whether computationally predicted AlphaFold2 structures perform comparably to experimental crystal structures, two crystal structures were selected for each target system following the criteria of Hahn et al. [9]. Specifically, one crystal structure per protein target was selected based on the recommended structure in Hahn et al., while a second structure was selected based on the Iridium score, which reflects experimental structure quality [9]. For Thrombin+, structure 2ZDA was selected due to its published status, as 2ZFF was not included in a publication. For BACE1+, structure 4DJY was selected due to its superior docking performance relative to other BACE1 structures reported by Hahn et al. [9]. BACE1 represents a particularly challenging case as it exhibits multiple conformational states, ligand-dependent binding modes, variable protonation states of binding site residues, and possible tautomeric forms of ligands [45].

**Fig. 4:**
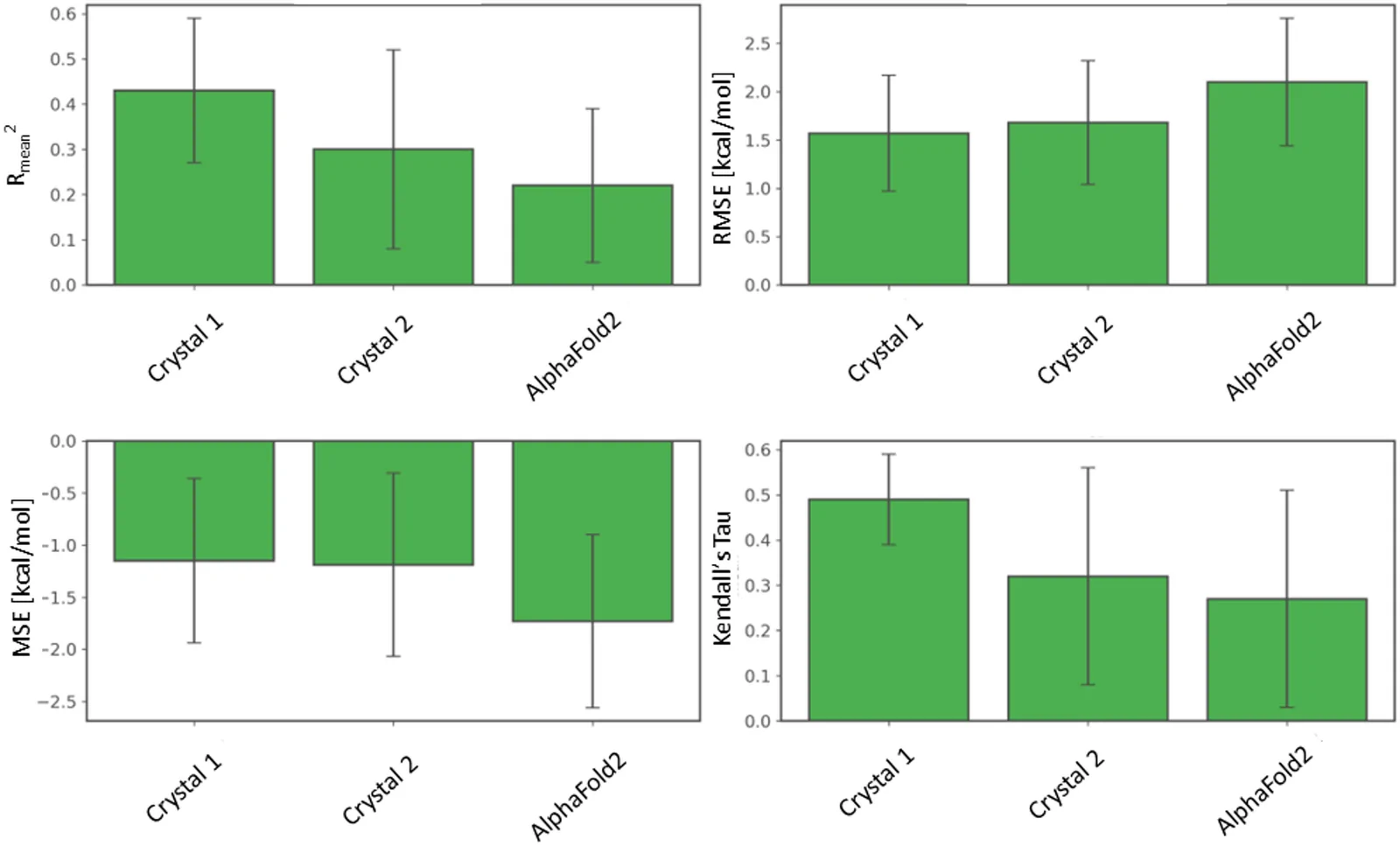
Comparison of experimental crystal structures and AlphaFold2 structures based on binding affinity results with docking for JACS Schrödinger+ dataset. For each protein target, the crystal structure with the highest R^2^ (Crystal 1) and the lowest R^2^ value (Crystal 2) in terms of correlation with experimental binding affinities is evaluated. The mean values and standard deviations of R^2^, RMSE, MSE and Kendall’s Tau of the binding scores are reported

Docking runs employed the Amber10:EHT force field, rigid docking and the GBVI scoring function. Figure 4 summarizes the averaged R_mean_^2^, RMSE_mean_ and MSE_mean_ and Kendall’s Tau_mean_ values across all targets for three structure sets: the crystal structures with the highest R^2^ (Crystal 1), those with lower R^2^ values (Crystal 2) and the AlphaFold2 models. The Crystal 1 set yielded the highest R_mean_^2^ value (0.43), while the AlphaFold2 structures performed worse than the Crystal 2 set, with R_mean_^2^ values of 0.22 and 0.30 respectively. Similarly, in terms of RMSE_mean_, the AlphaFold2 structures led to larger errors than the experimental structures (2.10 versus 1.57, for Crystal 1, and 1.68 kcal/mol, for Crystal 2). Further analysis, using the Kolmogorov-Smirnov test, did not detect a statistically significant difference at the selected significance threshold.

The scatter plots showing the correlation between the computationally predicted and the experimentally determined binding affinities, for each set of tested structures, are presented in Supplementary Fig. 2

### Accuracy of binding free energy calculations in each protein target

**Fig. 5:**
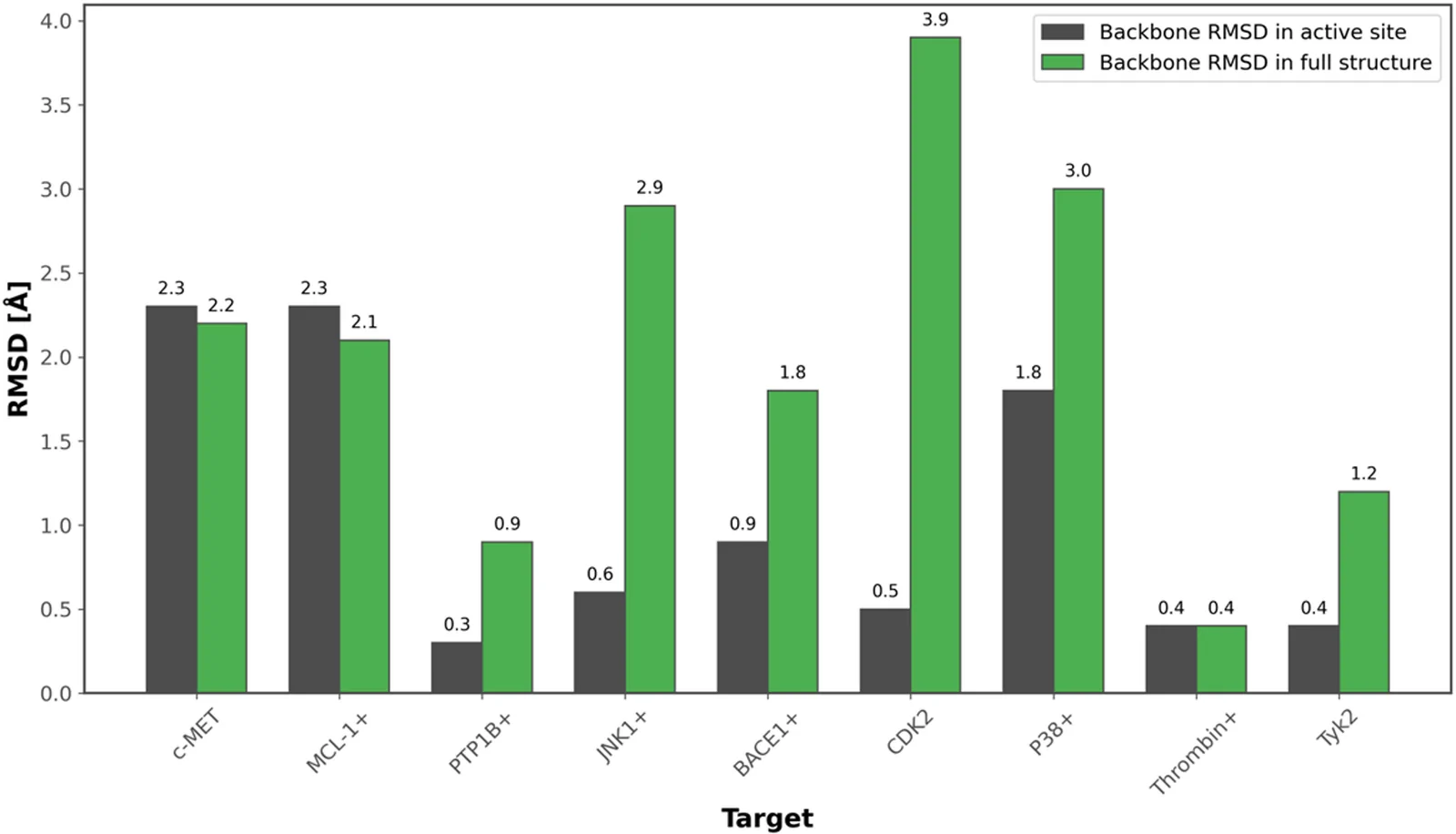
List of backbone RMSD values between AlphaFold2 models and the Crystal 1 structures of each target

To assess the effect of structure quality on docking performance, correlations were calculated between structure resolution and both R^2^ and RMSE values. These correlations were weak (0.14 for R^2^, 0.07 for RMSE), indicating that the conformational state of the crystal structure is more important than the resolution of the structure. Figure 5 lists the RMSD values between AlphaFold2 and Crystal 1 structures. Evaluation of the AlphaFold2 models revealed that for two of the nine protein targets (c-MET and MCL-1+), the backbone RMSD of the binding site residues atoms exceeded 2.0 Å compared to their respective Crystal 1 counterparts. This indicates a significant rearrangement of the binding pocket. For five targets (c-MET, CDK2, JNK1+, MCL-1+, and P38+), the backbone RMSD of the full structure exceeded 2.0 Å. This indicates that the long-range interactions might be altered compared to the crystal structure.

The correlation between full-structure RMSD and the difference in R^2^ between AlphaFold2 and Crystal 1 models was strongly negative (-0.81), whereas the correlation with RMSE differences was not as strong (-0.26). This suggests that the reduced performance of AlphaFold2 structures in docking arises primarily from deviations in binding pocket conformations.

Supplementary Tables 10 and 11 summarize the R^2^, RMSE, MSE and Kendall’s Tau values for each structure, including AlphaFold2 models, using rigid docking and the GBVI scoring function, based on the Amber10:EHT force field and the top docking pose.

### Effect of induced fit docking

To evaluate whether the reduced performance of AlphaFold2 models can be mitigated by optimizing the side-chain conformations, induced fit docking calculations were performed using the Amber10:EHT forcefield and GBVI scoring function across the nine protein targets of the JACS Schrödinger+ dataset. Accounting for receptor flexibility is critical in drug design, as ligand binding often induces conformational changes in the protein. Induced fit docking captures this adaptability, improving the representation of dynamic binding pockets [46].

**Fig. 6:**
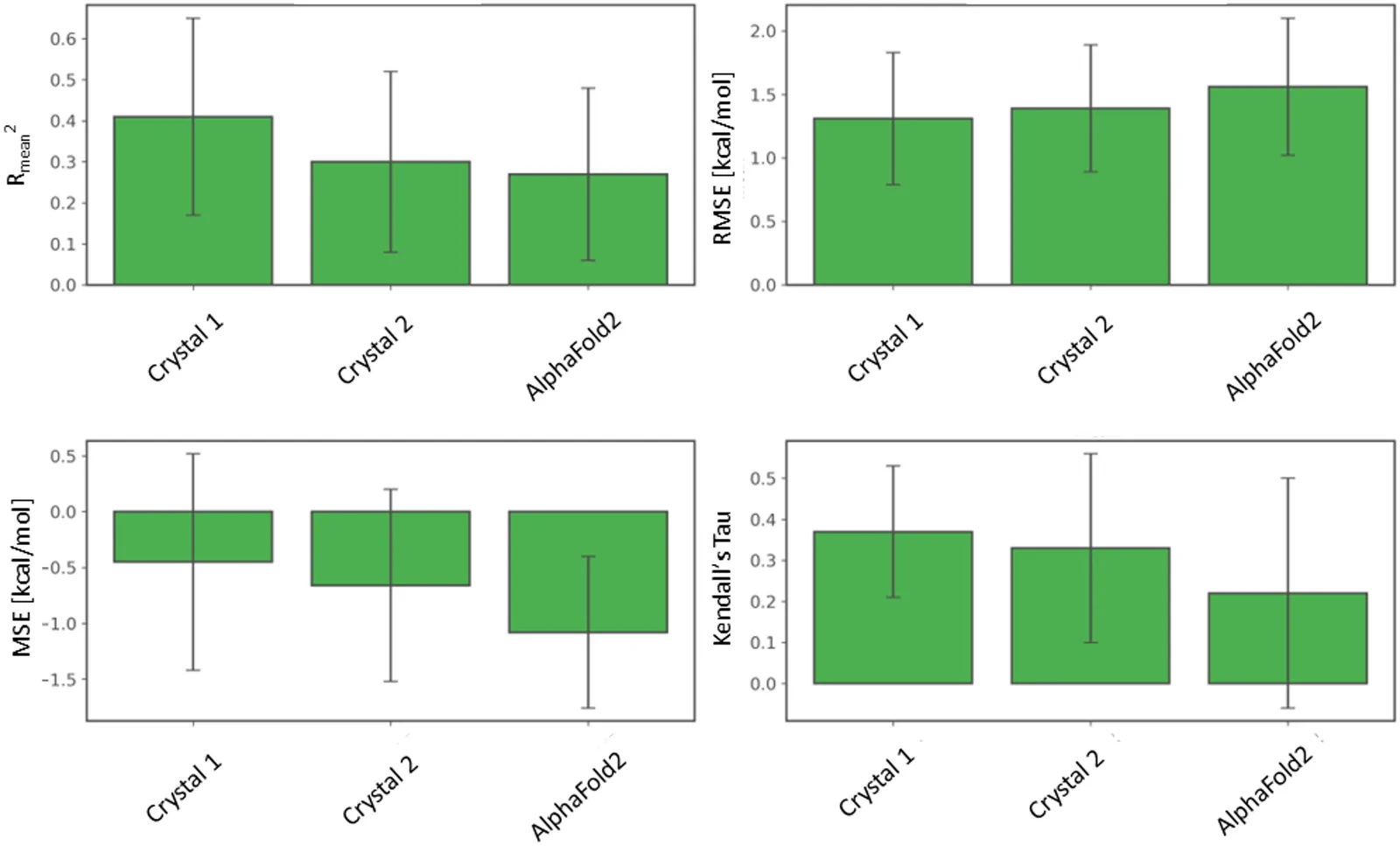
Comparison of binding scores results with induced fit docking using AlphaFold2 and experimental structures for the JACS Schrödinger+ dataset. For each protein target, the crystal structure with the highest R^2^ (Crystal 1) and the lowest R^2^ value (Crystal 2) in terms of correlation with experimental binding affinities is evaluated. The mean values and standard deviations of R^2^, RMSE, MSE and Kendall’s Tau of the binding scores are reported. The R^2^, RMSE, MSE and Kendall’s Tau values for each structure, including AlphaFold2 models, are summarized in Supplementary Fig. 4

The R_mean_^2^, RMSE_mean_ and MSE_mean_ and Kendall’s Tau_mean_ values across all systems (Fig. 6) showed that induced fit docking improved the R_mean_^2^ of docking with the AlphaFold2 structures from 0.22 to 0.27. Induced fit docking also reduced the RMSE_mean_ by ~ 0.3–0.5 kcal/mol for both crystal and AlphaFold structures. Systematic bias in predicted affinities was approximately halved.

### Consensus approach

To assess whether averaging docking results from different protein structures could reduce systematic errors, a consensus approach was applied using the optimized rigid docking protocol and the complete dataset. The error metrics were computed using average predicted binding scores across the crystal structures of each target for each ligand. The resulting values for R_mean_^2^, RMSE_mean_, MSE_mean_ and Kendall’s Tau_mean_ obtained through the optimized rigid docking protocol resulted in R_mean_^2^, RMSE_mean_, MSE_mean_ and Kendall’s Tau_mean_ values of 0.27, 1.71 kcal/mol, -1.20 kcal/mol and 0.33 respectively. Averaging the binding scores from both Crystal 1 and Crystal 2 structures yielded an R_mean_^2^ value nearly identical to that obtained using Crystal 2 alone. Similarly, RMSE_mean_ and MSE_mean_ values were comparable to those from the individual structures. These results indicate that the inclusion of lower-performing receptor structures in the averaging process does not improve binding affinity prediction accuracy within the context of this benchmark, and may even reduce overall performance.

### Performance of alternative docking tools and a molecular weight baseline

**Fig. 7:**
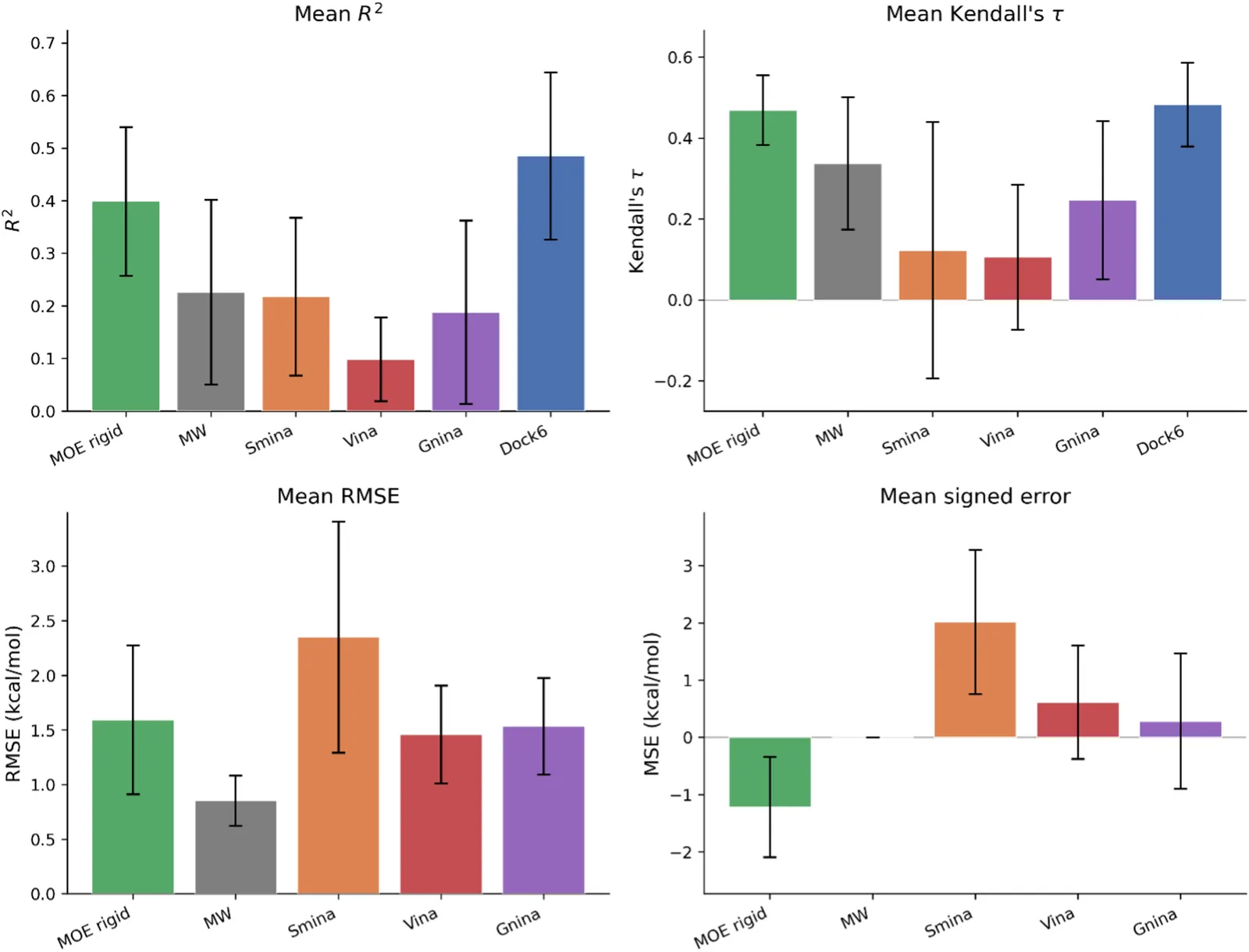
Comparison of affinity prediction performance for rigid MOE docking, a molecular weight regression baseline, Smina, AutoDock Vina, GNINA, and DOCK6 using the Crystal 1 receptor structures and the original JACS Schrödinger dataset of eight targets and 199 ligands. Bars represent unweighted means across the eight targets, and error bars represent standard deviations. The primary outputs were GBVI WSA dG for MOE, the Vina score for Smina and AutoDock Vina, CNNaffinity for GNINA, and the grid score for DOCK6. DOCK6 grid scores are not calibrated as an estimate of absolute binding free energy, so only R^2^ and Kendall’s τ are reported. The molecular weight regression (MW) was fitted separately for each target

To assess whether the observed performance was specific to MOE, we evaluated four additional docking programs, AutoDock Vina, Smina, GNINA, and DOCK6, using the Crystal 1 receptor structures and the ligand protonation and tautomeric states assigned during the MOE preparation workflow. The comparison was performed on the original JACS Schrödinger dataset with eight targets and 199 ligands. A target-specific linear regression based on ligand molecular weight was included as a baseline model. The results are summarized in Fig. 7 and Supplementary Table 17. Per-target results for the Crystal 1 MOE-ligand protocol are reported in Supplementary Tables 20–23. Mean results for the Crystal 1 and Crystal 2 Schrödinger-ligand protocols are given in Supplementary Tables 18 and 19, with the corresponding per-target values in Supplementary Tables 24–27 and 28–31, respectively.

The optimized rigid MOE protocol produced a mean R^2^ of approximately 0.40 and a mean Kendall’s τ of 0.47. Its mean RMSE and mean signed error were approximately 1.60 and − 1.22 kcal/mol, respectively. DOCK6 showed the strongest correlation and ranking performance among the evaluated docking programs, with a mean R^2^ of 0.49 ± 0.16 and a mean Kendall’s τ of 0.48 ± 0.10. Because the DOCK6 grid score is not calibrated to estimate absolute binding free energies, its RMSE and mean signed error were omitted.

The molecular weight regression (MW) produced a mean R^2^ of 0.23 ± 0.18 and a mean Kendall’s τ of 0.34 ± 0.16. This comparatively strong performance reflects an inherent bias of many congeneric series. Lead optimization frequently proceeds by adding or modifying substituents to improve potency, which can generate a strong correlation to molecular weight within a series. The results therefore indicate that the JACS Schrödinger benchmark contains a significant ligand-size dependent signal. This should not be interpreted as evidence that molecular weight is generally predictive of binding affinity, because increasing molecular weight does not necessarily increase potency. Rather, the molecular weight regression provides a useful null model for determining how much additional ranking information is contributed by the docking calculations.

The Vina-based workflows showed relatively modest average correlation with experimental affinities. Smina produced a mean R^2^ of 0.22 ± 0.15, comparable to the molecular weight baseline, whereas GNINA and AutoDock Vina yielded mean R^2^ values of 0.19 ± 0.17 and 0.10 ± 0.08, respectively. Their mean Kendall’s τ values were also lower than those obtained with MOE, DOCK6, and the molecular weight baseline. Smina showed the largest absolute and systematic errors, with a mean RMSE of 2.35 ± 1.06 kcal/mol and a mean signed error of + 2.02 ± 1.26 kcal/mol. Interestingly, AutoDock Vina and GNINA produced mean RMSE values of approximately 1.5 kcal/mol, despite their relatively weak correlation and ranking performance. The combination of low RMSE and weak correlation for AutoDock Vina and GNINA illustrates an important limitation of RMSE as an isolated measure of affinity prediction performance. The experimental binding free energy ranges within several benchmark targets are relatively narrow. Under these conditions, a method that predicts binding free energies close to the target-specific mean can achieve a low RMSE without reliably distinguishing more potent from less potent ligands. Such a model may therefore show apparently favorable absolute error while providing limited value for prioritization. Correlation, rank ordering, systematic error, and the experimental dynamic range should consequently be considered together when evaluating docking and more advanced binding affinity prediction methods.

To assess sensitivity with respect to receptor and ligand preparation, we compared Crystal 1 with MOE-assigned ligands (Fig. 7; Supplementary Table 17), Crystal 1 with the original Schrödinger ligands (Supplementary Table 18), and Crystal 2 with the original Schrödinger ligands (Supplementary Table 19). For the open source docking outputs, the effect was modest in most cases: Vina gave R^2^ values of about 0.09 to 0.19, Smina about 0.14 to 0.22, and GNINA about 0.10 to 0.22. DOCK6 was more receptor dependent, with R² of about 0.49 on Crystal 1 but only about 0.18 on Crystal 2. Notably, for MOE and DOCK6, the same receptor structures performed best. This is consistent with these specific structures capturing a more representative binding pocket state for this ligand series. For the programs with calibrated binding free energy outputs, RMSE remained in a relatively narrow range of about 1.5 to 2.6 kcal/mol, so RMSE alone should be interpreted cautiously when the experimental dynamic range is limited.

These results show that docking performance for binding affinity prediction depends strongly on the evaluation metric and the benchmark characteristics. MOE and DOCK6 provided the most useful ligand ranking in this benchmark, while MOE also produced a reasonable mean RMSE of approximately 1.5 kcal/mol. AutoDock Vina and GNINA achieved similarly low RMSE values but showed weak correlations with experimental binding affinities. The molecular weight regression produced the lowest RMSE of all evaluated approaches, largely because it was fitted separately to each target, but it also yielded stronger correlations with experiment than AutoDock Vina, Smina, and GNINA. This demonstrates that meaningful benchmarking requires careful assessment of dataset bias and simultaneous consideration of ranking performance, absolute error, systematic bias, and experimental affinity range.

## Conclusions

This study evaluated molecular docking protocols for the prediction of binding affinities using an extended JACS Schrödinger benchmark set. Several parameters were investigated, including the scoring function, force field, the use of multiple docking poses, protein structure, and receptor flexibility. Among all parameters tested, the scoring function and the protein structure had the greatest influence on binding affinity prediction accuracy. The GBVI WSA dG scoring function provided the best performance for the selected two targets, with an average R² of 0.41, while the Affinity dG scoring function achieved an R² of 0.17. These observations are consistent with the results of Cheng et al., who showed that only a few scoring functions achieve significant correlations with experimental binding affinities [47]. The choice of force field (Amber10:EHT or MMFF94x) had little impact on R², although Amber10:EHT produced slightly lower RMSE and MSE values, indicating marginally higher binding affinity accuracy. Using multiple docking poses to represent ligand conformational flexibility led to only minor improvement over using the top pose, with R² increasing from 0.32 to 0.34.

The quality of the protein structure was found to be a critical factor. The Crystal 1 structure set produced the highest mean R² value of approximately 0.43, while Crystal 2 structures and AlphaFold2 models yielded mean R² values of 0.30 and 0.22, respectively. No correlation was observed between the crystallographic resolution and binding affinity prediction accuracy, suggesting that the conformational state is more important than resolution. Structural analysis showed that docking using AlphaFold2 models frequently produced inaccurate poses, with RMSD values exceeding 2 Å for many ligands. These results are in agreement with previously reported studies, which have shown that side-chain inaccuracies, conformational bias, and low-confidence regions in AlphaFold2 models reduce their reliability for docking [48–53]. Rovira et al., using Glide docking, cautioned against overreliance on AlphaFold2 models for virtual screening [48]. Their work focused on a diverse set of 10 targets, for which two AlphaFold2 models of different maximum sequence similarity were included and compared to crystal structures. Similarly to our findings, their results indicated significantly higher ligand RMSD values in the docked complexes of AlphaFold2 models, compared to those of crystal structures. They highlighted that AlphaFold2 models tend to display apo binding sites, with structural deviation compared to the holo structures. Moreover, they identified differences on the side chain level, compared to the crystal structures, that require post modelling optimisation, in order to improve the model.

Similarly, Holcomb et al. observed significant discrepancies in redocking experiments, similar to our observations, where AlphaFold2 models frequently led to misdocked ligands, which were docked either in incorrect binding pockets or in the correct ones but in erroneous orientations (60% versus 34% of crystal structures for RMSD > 5 Å, whereas 17% versus 41% for RMSD < 2 Å) [50]. In addition to incorporating side-chain flexibility during docking, it was also argued that removing low confidence regions could improve the performance of AlphaFold2 structures.

Deep-learning docking methods based on AlphaFold2 structures were also outperformed by classical docking [54]. The use of induced-fit docking to account for receptor flexibility improved the performance of AlphaFold2 models slightly, with R² increasing from 0.22 to 0.27 and RMSE decreasing from 2.10 to 1.56 kcal/mol.

**Fig. 8:**
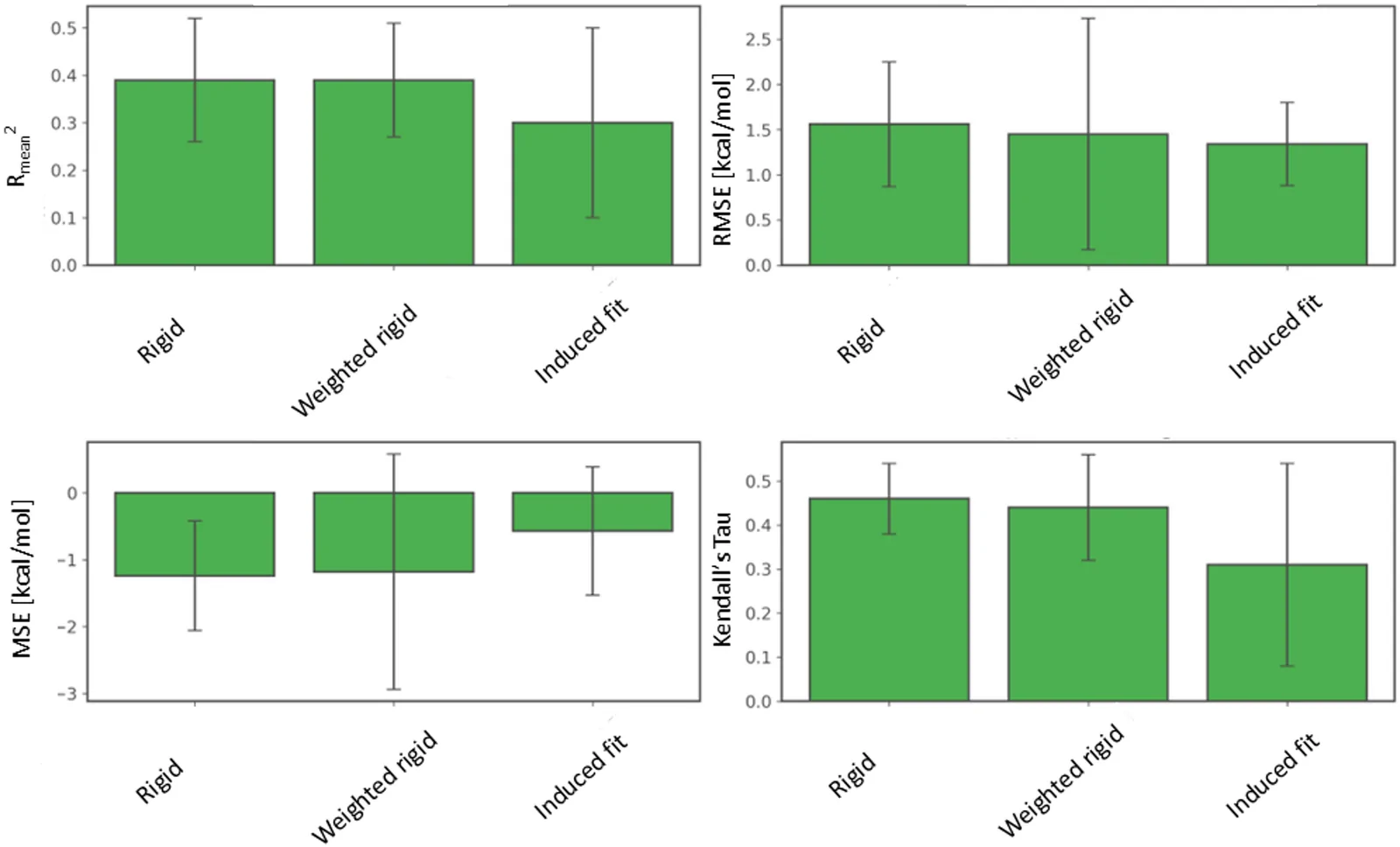
Average performance metrics using the optimized docking protocol for the original JACS Schrödinger dataset (8 protein targets and 199 ligands) by Wang et al. [14], using the Crystal 1 structure, GBVI WSA dG scoring function, TOP1 pose and Amber10:EHT force field with rigid and induced fit docking. “Weighted rigid” weights each target by the number of ligands

The results of the optimized docking protocol for the original JACS Schrödinger dataset are summarized in Fig. 8. The corresponding results for the individual targets are presented in Supplementary Tables 13 and Supplementary Fig. 5. The calculations used the Crystal 1 structures, the GBVI WSA dG scoring function, the Amber10:EHT force field, and the top ranked docking pose. For the rigid docking protocol, the R_mean_^2^ was 0.39 and the RMSE_mean_ 1.56 kcal/mol. For the induced-fit docking protocol, the corresponding values were 0.30 and 1.34 kcal/mol. Although induced-fit docking produced a lower correlation with experiment, it improved the RMSE and MSE values, indicating greater accuracy in the predicted absolute binding affinities.

A detailed comparison with advanced computational approaches was reported in Reference 55, using the four-target subset (TYK2, CDK2, JNK1, and P38) from the original JACS Schrödinger dataset [55]. For this subset, based on identical PDB structures as used here, absolute binding free energy (ABFE) calculations produced the highest mean R² value of 0.56, representing the gold standard for binding affinity calculations [55–56]. The Boltz-2 cofolding model achieved a mean R² value of 0.38. Additional methods reported in the same work included the fragment molecular orbital (FMO) approach (R² = 0.30), the Chemgauss4 docking score (R² = 0.07) and MM/PBSA simulations (R² = 0.03) [55]. Using the same targets and protein structures, the optimized docking protocol for DOCK6 and MOE reach mean R² of 0.43 and 0.30, which is comparable to Boltz-2 cofolding and FMO, and higher than Chemgauss4 and MM/PBSA. The main difference between the published results and the present results are the structure preparation procedures and binding modes. When using the original ligand structures from the Schrödinger benchmark in DOCK6, the mean R² remains 0.44, which indicates that the ligand preparation does not have a significant impact on this subset. Therefore, the results indicate that, under optimized conditions, molecular docking can provide a computationally efficient baseline for binding affinity prediction, within the range reported for several computationally more demanding methods. However, this comparison is complicated by differences in structure preparation and evaluation procedures.

Comparisons with relative binding free energy (RBFE) methods, which were assessed using the original JACS Schrödinger dataset, also indicate that binding affinity predictions from docking perform better than previously expected [15, 57]. RBFE calculations employing the OpenFF1.0, GAFF2.1x, and OPLS3e force fields produced RMSE values of 1.21, 1.11, and 1.14 kcal/mol, respectively. The RBFE calculations based on CGenFF achieved an RMSE of 1.35 kcal/mol, which is almost identical with the RMSE of 1.34 kcal/mol obtained for induced-fit docking in Fig. 8. Notably, relative methods, such as RBFE, often benefit from partial cancellation of systematic errors between similar ligands, which can artificially increase the apparent binding affinity prediction accuracy. Docking, in contrast, estimates absolute binding affinities and does not benefit from this effect. Given its substantially lower computational cost, the level of predictive performance obtained with docking is noteworthy and supports its continued use as a baseline method for affinity prediction.

Docking is most often evaluated by pose reproduction, as illustrated by community blind challenges such as D3R and CASP, where ligand pose prediction remains a central benchmark and receptor quality strongly affects performance [58, 59]. Here, we focused on binding affinity prediction instead, to compare optimized docking directly with more computationally demanding methods. On this benchmark, the optimized DOCK6 and MOE protocols achieved correlation coefficients similar to those reported for certain MM/PBSA, FMO, and Boltz2 implementations evaluated on the same target subset. The results do not establish the superiority of docking. However, if methods that include sampling, solvation, more accurate interaction energies, or entropic effects more explicitly do not substantially outperform docking, this should raise questions about hidden dataset biases, the structural preparation, or the implementation of those methods. For this reason, new computational approaches should be benchmarked against well-optimized docking protocols and should demonstrate clear improvements before adoption in real-world drug discovery pipelines.

## Supplementary Information

Below is the link to the electronic supplementary material.


Supplementary Material 1


## Data Availability

No datasets were generated or analysed during the current study. All underlying data are available in the article itself and its Supporting Information.
