# Supplementary material for: Evaluating molecular docking for binding affinity predictions: a systematic analysis of key parameters and the utility of AlphaFold2 structures for the Schrödinger dataset
Source: J Comput Aided Mol Des. 2026 Aug 29;40(1):219. doi: 10.1007/s10822-026-00929-9 (PMC13526069; doi:10.1007/s10822-026-00929-9)
Supplement: Supplementary file 1 [file 10822_2026_929_MOESM1_ESM.docx]

Supplementary Information

Additional technical details regarding structure preparation and docking protocols are provided in this section for reproducibility. Hydrogen atoms are added to protein structures lacking experimental hydrogen coordinates according to standard valence rules and the protonation/tautomeric states assigned by Protonate 3D with default pH 7.0 settings. Initial hydrogen positions are generated consistently with the selected protonation states. The resulting all-atom structure is then subjected to energy minimization in the presence of implicit solvent model (Generalized Born / Volume Integral), allowing hydrogen coordinates to relax within the local electrostatic environment. During this refinement, hydrogen atoms reorient to optimize intramolecular hydrogen bonding and interactions with solvent. Missing residues were reconstructed using the Correct option in Structure Preparation, and missing loops were modelled using the Loop Modeler algorithm. The crystal structures of the Thrombin target were docked without a modelled loop, as its implementation negatively affected the binding affinity prediction accuracy. Ligand structures were energy-minimized using the selected force field and partial charges recalculated prior to docking. For the BACE1 target, both possible monoprotonated states of the catalytic Asp residues were considered, in combination with the two tautomeric forms of each ligand, in order to select the state of the Asp residues in the active site of the studied structures and the form of the ligands.

Docking was performed using the Dock function in MOE 2022.03. Binding-site residues were defined using the Selected Residues option, encompassing those involved in direct ligand contacts and pocket shaping. The Triangle Matcher placement method was employed to generate up to 1000 poses per ligand in rigid docking and 250 poses per ligand for induced-fit docking. Induced-fit docking was performed using the Free option, allowing side-chain flexibility for residues within 6 Å of the ligand, while maintaining a fixed protein backbone. Side chains in the selected region were treated as fully flexible during docking.

Two force fields, available in MOE, were tested: MMFF94x, an all-atom force field parameterized for small organic molecules, and Amber10:EHT, which combines the Amber10 protein force field with an extended Hückel treatment of small molecules. The scoring functions were evaluated as follows:

1. GBVI WSA dG, a physics-based, force-field-dependent scoring function trained on the SIE dataset of 99 protein-ligand complexes, calculated according to

$$\Delta G\approx c+\alpha\left[ \frac{2}{3}\left( \Delta E_{Cou}+\Delta E_{sol}+\Delta E_{Vdw}+\beta\Delta ESA_{weighted} \right) \right]$$

where c represents the average change in rotational and translational entropy. The constants α, β, and c are determined during training and depend on the force field. E_Coul_ is the Coulombic electrostatic term calculated using the currently loaded charges with a constant dielectric of ε_ι_ = 1. E_sol_ is the solvation electrostatic term calculated using the GB/VI solvation model. E_vdW_ represents the van der Waals contribution to binding. SA_weighted_ is the surface area weighted by exposure, penalizing exposed surface area.

2. Affinity dG, a semiempirical linear scoring function estimating enthalpic contributions to binding via atomic interaction terms:

$$\Delta G=C_{hb}f_{hb}+C_{ion}f_{ion}+C_{mlig}f_{mlig}+C_{hh}f_{hh}+C_{hp}f_{hp}+C_{aa}f_{aa}$$

where the f terms count specific atomic contacts fractionally, and the C terms are coefficients weighting these contributions. hb refers to hydrogen bond donor-acceptor interactions, ion to ionic interactions evaluated using a Coulomb-like term, mlig to metal ligation interactions, hh to favorable hydrophobic interactions, hp to unfavorable interactions between hydrophobic and polar atoms, and aa to weak, generally favorable interactions between any two atoms.

Docked poses were ranked according to their final rescoring value, and binding energies were extracted either from the top-ranked pose or as an average over the five best-scoring poses. The pose quality of the optimized rigid docking protocol was assessed via heavy-atom RMSD between the docked ligand and its crystallographic position, calculated using CalcLigRMSD.

**Supplementary Table 1**. List of number of ligands, minimum, maximum and range of experimental binding affinities in each target.

| Target | Number of ligands | ΔG_min_ [kcal/mol] | ΔG_max_ [kcal/mol] | ΔG_range_ [kcal/mol] |
| --- | --- | --- | --- | --- |
| Thrombin+ | 12 | -11.02 | -7.48 | 3.54 |
| c-MET | 24 | -12.28 | -6.17 | 6.11 |
| CDK2 | 16 | -11.25 | -7.04 | 4.21 |
| JNK1+ | 59 | -10.67 | -6.79 | 3.88 |
| MCL-1+ | 47 | -9.96 | -5.78 | 4.18 |
| P38+ | 35 | -12.26 | -8.48 | 3.78 |
| PTP1B+ | 26 | -12.46 | -7.34 | 5.12 |
| Tyk2 | 16 | -11.74 | -7.44 | 4.30 |
| BACE1+ | 43 | -11.28 | -6.82 | 4.46 |

**Effect of scoring function**

Differences were observed, for the majority of the protein targets, with respect to the scoring functions. For 3DDQ structure in CDK2, the GBVI scoring function yielded an R^2^ value of 0.22, whereas for Affinity dG the respective value was 0.00. Similarly, the AlphaFold2 showed a decrease of 0.28 for the R^2^. The structures of the JNK1+ target showed increased RMSE and MSE values for Affinity dG, compared to GBVI (RMSE < 2 for GBVI versus RMSE > 2 for Affinity dG and MSE < 1.5 for GBVI versus MSE > 2 kcal/mol for Affinity dG). In the case of MCL-1+, the GBVI provided R^2^ ~ 0.55, RMSE < 1 and MSE < 0.5 for both crystal structures, whereas for Affinity dG the respective values were ~ 0.05, RMSE > 1.5 and MSE > 1 kcal/mol. For P38+, the values for R^2^ in the GBVI scoring function were higher than the Affinity dG in the crystal structures (~0.35 versus ~ 0.10), while the respective RMSE and MSE values much lower (RMSE/MSE < 3 for GBVI versus RMSE/MSE > 3 kcal/mol for Affinity dG). Similar results to P38+ were obtained for PTP1B+, Tyk2 and BACE1+.

**Supplementary Table 2**. List of R^2^, RMSE, MSE and Kendall’s Tau values for the GBVI WSA dG scoring function, using the binding score with rigid docking, Amber10:EHT force field and lowest binding affinity pose of all structures in each target.

| Target | Structure | Scoring function | | |  |
| --- | --- | --- | --- | --- | --- |
|  |  | GBVI WSA dG | | |  |
|  |  | R^2^ | RMSE [kcal/mol] | MSE [kcal/mol] | Kendall’s Tau |
| Thrombin+ | 2ZDA | 0.41 | 0.98 | -0.54 | 0.52 |
|  | 5JZY | 0.10 | 0.85 | -0.07 | 0.24 |
|  | AlphaFold2 | 0.00 | 0.97 | -0.38 | 0.33 |
| c-MET | 5EOB | 0.70 | 1.40 | -0.63 | 0.69 |
|  | 4R1Y | 0.69 | 1.40 | -0.62 | 0.70 |
|  | AlphaFold2 | 0.50 | 2.18 | -1.73 | 0.51 |
| CDK2 | 3DDQ | 0.22 | 1.62 | -1.22 | 0.43 |
|  | 1H1Q | 0.13 | 1.90 | -1.54 | 0.27 |
|  | AlphaFold2 | 0.36 | 2.05 | -1.79 | 0.40 |
| JNK1+ | 3ELJ | 0.49 | 1.31 | -1.02 | 0.53 |
|  | 2GMX | 0.48 | 1.68 | -1.46 | 0.51 |
|  | AlphaFold2 | 0.47 | 1.53 | -1.28 | 0.52 |
| MCL-1+ | 4WMU | 0.63 | 0.88 | -0.36 | 0.59 |
|  | 4HW3 | 0.45 | 0.91 | 0.01 | 0.46 |
|  | AlphaFold2 | 0.21 | 2.40 | -2.17 | 0.37 |
| P38+ | 3FLN | 0.36 | 2.26 | -2.12 | 0.44 |
|  | 3FLY | 0.30 | 2.54 | -2.40 | 0.37 |
|  | AlphaFold2 | 0.21 | 3.34 | -3.22 | 0.29 |
| PTP1B+ | 2QBS | 0.49 | 0.91 | -0.06 | 0.52 |
|  | 2HB1 | 0.48 | 1.02 | -0.42 | 0.46 |
|  | AlphaFold2 | 0.02 | 1.62 | -0.67 | 0.14 |
| Tyk2 | 4GIH | 0.20 | 2.59 | -2.30 | 0.33 |
|  | 3LXP | 0.04 | 2.56 | -2.19 | -0.10 |
|  | AlphaFold2 | 0.06 | 2.81 | -2.49 | -0.32 |
| BACE1+ | 4DJY | 0.38 | 2.21 | -2.10 | 0.39 |
|  | 4DJW | 0.02 | 2.22 | -2.04 | 0.00 |
|  | AlphaFold2 | 0.15 | 2.01 | -1.85 | -0.20 |

**Supplementary Table 3.** List of R^2^, RMSE, MSE and Kendall’s Tau values for the Affinity dG function, using the binding score with rigid docking, Amber10:EHT force field and lowest binding affinity pose of all structures in each target.

| Target | Structure | Scoring function | | |  |
| --- | --- | --- | --- | --- | --- |
|  |  | Affinity dG | | |  |
|  |  | R^2^ | RMSE [kcal/mol] | MSE [kcal/mol] | Kendall’s Tau |
| Thrombin+ | 2ZDA | 0.56 | 1.20 | -1.01 | 0.36 |
|  | 5JZY | 0.02 | 1.05 | -0.03 | -0.18 |
|  | AlphaFold2 | 0.00 | 1.24 | -0.77 | -0.06 |
| c-MET | 5EOB | 0.10 | 2.30 | -1.63 | 0.21 |
|  | 4R1Y | 0.03 | 2.38 | -1.69 | 0.08 |
|  | AlphaFold2 | 0.35 | 2.46 | -1.98 | 0.37 |
| CDK2 | 3DDQ | 0.00 | 1.86 | -1.37 | 0.10 |
|  | 1H1Q | 0.20 | 2.16 | -1.88 | 0.27 |
|  | AlphaFold2 | 0.08 | 1.36 | -0.69 | 0.20 |
| JNK1+ | 3ELJ | 0.52 | 2.56 | -2.39 | 0.55 |
|  | 2GMX | 0.40 | 3.34 | -3.20 | 0.43 |
|  | AlphaFold2 | 0.39 | 3.07 | -2.91 | 0.41 |
| MCL-1+ | 4WMU | 0.04 | 1.68 | 1.20 | 0.09 |
|  | 4HW3 | 0.05 | 2.15 | 1.78 | 0.08 |
|  | AlphaFold2 | 0.01 | 2.20 | -1.88 | 0.03 |
| P38+ | 3FLN | 0.09 | 3.21 | -3.04 | 0.24 |
|  | 3FLY | 0.06 | 3.27 | -3.09 | 0.20 |
|  | AlphaFold2 | 0.13 | 3.38 | -3.23 | 0.28 |
| PTP1B+ | 2QBS | 0.24 | 2.59 | -2.35 | 0.33 |
|  | 2HB1 | 0.24 | 2.50 | -2.24 | 0.33 |
|  | AlphaFold2 | 0.26 | 3.18 | -2.99 | 0.35 |
| Tyk2 | 4GIH | 0.03 | 4.09 | -3.85 | -0.05 |
|  | 3LXP | 0.03 | 3.97 | -3.75 | -0.22 |
|  | AlphaFold2 | 0.02 | 3.91 | -3.70 | 0.03 |
| BACE1+ | 4DJY | 0.01 | 2.76 | -2.59 | -0.19 |
|  | 4DJW | 0.01 | 2.11 | -1.85 | -0.16 |
|  | AlphaFold2 | 0.02 | 1.38 | -0.74 | -0.20 |

With regard to the extended analysis of Thrombin+ and c-MET, the 5JZY structure (R_mean_^2^ of 0.28 versus 0.01) and the AlphaFold2 structure of Thrombin+ (R_mean_^2^ of 0.14 versus 0.05) yielded higher values for GBVI scoring than Affinity dG, while the 2ZDA structure of Thrombin+ indicated that Affinity dG performs better than GBVI (R_mean_^2^ of 0.52 versus 0.27). The RMSE_mean_ exhibited differences of about 0.2-0.5 kcal/mol between GBVI and Affinity scoring, indicating that GBVI performs better. The MSE_mean_ showed a maximum difference of 0.65 kcal/mol for the 2ZDA structure in Thrombin+, and smaller differences in 5JZY and AlphaFold2 structures. For c-MET, the GBVI scoring function yielded higher R_mean_^2^ values for all structures than Affinity dG. Both 4R1Y and 5EOB showed a R_mean_^2^ value of ~0.70, while the R_mean_^2^ of the AlphaFold2 structure was lower with 0.47. On the other hand, the Affinity scoring function only yielded R_mean_^2^ values between 0.05 and 0.29. In addition, the RMSE_mean_ values were lower with GBVI (between 1.4 and 2.3 kcal/mol).

**Supplementary Table 4.** Comparison of the GBVI WSA dG scoring function and Affinity dG scoring functions based on the mean square of the Pearson correlation coefficient (R_mean_^2^) values of binding scores from rigid docking in Thrombin+ and c-MET. Mean R^2^ values are listed for each of the protein structures. The mean values and standard deviations of R^2^ are listed for each protein target.

| Structure | Scoring function / R_mean_^2^ | |
| --- | --- | --- |
|  | GBVI WSA dG | Affinity dG |
| 5JZY | 0.28 | 0.01 |
| 2ZDA | 0.27 | 0.52 |
| AlphaFold2 | 0.14 | 0.05 |
| Average over Thrombin+’s structures $\pm$ Standard deviation | 0.23 $\pm$ 0.16 | 0.19 $\pm$ 0.23 |
| 5EOB | 0.69 | 0.05 |
| 4R1Y | 0.64 | 0.09 |
| AlphaFold2 | 0.47 | 0.29 |
| Average over c-MET’s structures $\pm$ Standard deviation | 0.60 $\pm$ 0.10 | 0.14 $\pm$ 0.12 |

**Supplementary Table 5.** Comparison of the GBVI WSA dG scoring function and Affinity dG scoring function based on the mean root mean squared error (RMSE_mean_) of the binding for rigid docking in Thrombin+ and c-MET. Mean RMSE values and standard deviations are reported for each protein structure and for each target.

| Structure | Scoring function / RMSE_mean_ [kcal/mol] | |
| --- | --- | --- |
|  | GBVI WSA dG | Affinity dG |
| 5JZY | 0.83 | 1.05 |
| AlphaFold2 | 0.96 | 1.39 |
| 2ZDA | 1.04 | 1.45 |
| Average over Thrombin+’s structures $\pm$ Standard deviation | 0.94 $\pm$ 0.09 | 1.29 $\pm$ 0.24 |
| 4R1Y | 1.41 | 2.42 |
| 5EOB | 1.44 | 2.45 |
| AlphaFold2 | 2.32 | 2.57 |
| Average over c-MET’s structures $\pm$ Standard deviation | 1.72 $\pm$ 0.43 | 2.48 $\pm$ 0.13 |

**Supplementary Table 6.** Comparison of the GBVI WSA dG scoring function and Affinity dG scoring function based on the mean signed error (MSE_mean_) of the binding scores for rigid docking in Thrombin+ and c-MET. Mean MSE values and standard deviations are reported for each protein structure and for each target.

| Structure | Scoring function / MSE_mean_ [kcal/mol] | |
| --- | --- | --- |
|  | GBVI WSA dG | Affinity dG |
| 5JZY | -0.21 | -0.23 |
| AlphaFold2 | -0.46 | -0.95 |
| 2ZDA | -0.62 | -1.27 |
| Average over Thrombin+’s structures $\pm$ Standard deviation | -0.43 $\pm$ 0.19 | -0.82 $\pm$ 0.48 |
| 4R1Y | -0.55 | -1.78 |
| 5EOB | -0.67 | -1.79 |
| AlphaFold2 | -1.88 | -2.08 |
| Average over c-MET’s structures $\pm$ Standard deviation | -1.03 $\pm$ 0.61 | -1.88 $\pm$ 0.19 |

**Supplementary Table 7.** Comparison of the GBVI WSA dG scoring function and Affinity dG scoring function based on the mean Kendall’s Tau (Kendall’s Tau_mean_) values of the binding scores for rigid docking in Thrombin+ and c-MET. Kendall’s Tau_mean_ values and standard deviations are reported for each protein structure and for each target.

| Structure | Scoring function / Kendall’s Tau_mean_ | |
| --- | --- | --- |
|  | GBVI WSA dG | Affinity dG |
| AlphaFold2 | 0.46 | -0.12 |
| 2ZDA | 0.40 | 0.39 |
| 5JZY | 0.29 | -0.05 |
| Average over Thrombin+’s structures $\pm$ Standard deviation | 0.38 $\pm$ 0.13 | 0.08 $\pm$ 0.24 |
| 5EOB | 0.68 | 0.13 |
| 4R1Y | 0.67 | 0.17 |
| AlphaFold2 | 0.50 | 0.39 |
| Average over c-MET’s structures $\pm$ Standard deviation | 0.61 $\pm$ 0.09 | 0.23 $\pm$ 0.13 |

**Effect of force field**

Amber10:EHT yielded higher R_mean_^2^ values in five out of nine protein targets (c-MET, CDK2, MCL-1+, P38+, PTP1B+) and lower values in three (Thrombin+, Tyk2, and BACE1+). However, in the cases of both Tyk2 and BACE1+, the resulting RMSE_mean_ and MSE_mean_ values of MMFF94x were larger compared to the respective ones in Amber10:EHT. For JNK1+, both force fields yielded a R_mean_^2^ value of 0.48, but the RMSE_mean_ and MSE_mean_ values of Amber10:EHT were smaller compared to MMFF94x (1.51 versus 2.01 kcal/mol and -1.26 versus -1.83 kcal/mol respectively).

A)

B)

C)

D)

**Supplementary Figure 1.** Average values of R^2^, RMSE, MSE and Kendall’s Tau values per protein target based on rigid docking and the GBVI scoring function, for the force fields Amber10:EHT and MMFF94x. A) R^2^, B) RMSE, C) MSE and D) Kendall’s Tau.

**Supplementary Table 8**. List of R^2^, RMSE, MSE and Kendall’s Tau values for the Amber10:EHT force field, using the binding score of each structure with rigid docking, GBVI scoring function, and lowest binding affinity pose.

| Target | Structure | Force field | | |  |
| --- | --- | --- | --- | --- | --- |
|  |  | Amber10:EHT | | |  |
|  |  | R^2^ | RMSE [kcal/mol] | MSE [kcal/mol] | Kendall’s Tau |
| Thrombin | **2ZDA** | **0.41** | **0.98** | **-0.54** | **0.52** |
|  | 5JZY | 0.10 | 0.85 | -0.07 | 0.24 |
|  | AlphaFold2 (Thrombin+) | 0.01 | 0.97 | -0.38 | 0.33 |
| c-MET | 4R1Y | 0.69 | 1.40 | -0.62 | 0.70 |
|  | **5EOB** | **0.70** | **1.40** | **-0.63** | **0.69** |
|  | AlphaFold2 (c-MET) | 0.50 | 2.18 | -1.73 | 0.51 |
| CDK2 | 1H1Q | 0.13 | 1.90 | -1.54 | 0.27 |
|  | 3DDQ | 0.22 | 1.62 | -1.22 | 0.43 |
|  | **AlphaFold2 (CDK2)** | **0.36** | **2.05** | **-1.79** | **0.40** |
| JNK1+ | 2GMX | 0.48 | 1.68 | -1.46 | 0.51 |
|  | **3ELJ** | **0.49** | **1.31** | **-1.02** | **0.53** |
|  | AlphaFold2 (JNK1+) | 0.47 | 1.53 | -1.28 | 0.52 |
| MCL-1+ | 4HW3 | 0.45 | 0.91 | 0.01 | 0.46 |
|  | **4WMU** | **0.63** | **0.88** | **-0.36** | **0.59** |
|  | AlphaFold2 (MCL-1+) | 0.21 | 2.40 | -2.17 | 0.37 |
| P38+ | **3FLN** | **0.36** | **2.26** | **-2.12** | **0.44** |
|  | 3FLY | 0.30 | 2.54 | -2.40 | 0.37 |
|  | AlphaFold2 (P38+) | 0.21 | 3.34 | -3.22 | 0.29 |
| PTP1B+ | 2HB1 | 0.48 | 1.02 | -0.42 | 0.46 |
|  | **2QBS** | **0.49** | **0.91** | **-0.06** | **0.52** |
|  | AlphaFold2 (PTP1B+) | 0.02 | 1.62 | -0.67 | 0.14 |
| Tyk2 | 3LXP | 0.04 | 2.56 | -2.19 | -0.10 |
|  | **4GIH** | **0.20** | **2.59** | **-2.30** | **0.33** |
|  | AlphaFold2 (Tyk2) | 0.06 | 2.81 | -2.49 | -0.32 |
| BACE1+ | **4DJY** | **0.38** | **2.21** | **-2.10** | **0.39** |
|  | 4DJW | 0.02 | 2.22 | -2.04 | 0.00 |
|  | AlphaFold2 (BACE1+) | 0.15 | 2.01 | -1.85 | 0.16 |

**Supplementary Table 9.** List of R^2^, RMSE, MSE and Kendall’s Tau values for the MMFF94x force field, using the binding score of each structure with rigid docking, GBVI scoring function, and lowest binding affinity pose.

| Target | Structure | Force field | | |  |
| --- | --- | --- | --- | --- | --- |
|  |  | MMFF94x | | |  |
|  |  | R^2^ | RMSE [kcal/mol] | MSE [kcal/mol] | Kendall’s Tau |
| Thrombin+ | 2ZDA | 0.13 | 1.09 | -0.69 | 0.27 |
|  | **5JZY** | **0.45** | **0.81** | **-0.34** | **0.33** |
|  | AlphaFold2 (Thrombin+) | 0.27 | 0.95 | -0.54 | 0.58 |
| c-MET | 4R1Y | 0.58 | 1.42 | -0.48 | 0.63 |
|  | **5EOB** | **0.69** | **1.48** | **-0.71** | **0.66** |
|  | AlphaFold2 (c-MET) | 0.44 | 2.47 | -2.04 | 0.48 |
| CDK2 | 1H1Q | 0.03 | 3.09 | -2.83 | -0.07 |
|  | 3DDQ | 0.09 | 2.33 | -2.05 | 0.32 |
|  | **AlphaFold2 (CDK2)** | **0.36** | **3.36** | **-3.20** | **0.43** |
| JNK1+ | 2GMX | 0.48 | 2.19 | -2.02 | 0.51 |
|  | 3ELJ | 0.46 | 1.86 | -1.66 | 0.50 |
|  | **AlphaFold2 (JNK1+)** | **0.49** | **1.97** | **-1.79** | **0.54** |
| MCL-1+ | 4HW3 | 0.27 | 1.01 | -0.32 | 0.33 |
|  | **4WMU** | **0.66** | **1.02** | **-0.70** | **0.57** |
|  | AlphaFold2 (MCL-1+) | 0.17 | 2.79 | -2.59 | 0.38 |
| P38+ | **3FLN** | **0.32** | **2.41** | **-2.27** | **0.40** |
|  | 3FLY | 0.22 | 2.86 | -2.72 | 0.35 |
|  | AlphaFold2 (P38+) | 0.28 | 3.88 | -3.78 | 0.35 |
| PTP1B+ | 2HB1 | 0.36 | 1.20 | -0.65 | 0.40 |
|  | **2QBS** | **0.52** | **0.91** | **-0.22** | **0.54** |
|  | AlphaFold2 (PTP1B+) | 0.09 | 2.81 | -2.47 | 0.39 |
| Tyk2 | 3LXP | 0.00 | 3.10 | -2.82 | 0.02 |
|  | **4GIH** | **0.69** | **2.70** | **-2.48** | **0.65** |
|  | AlphaFold2 (Tyk2) | 0.14 | 3.26 | -3.04 | 0.20 |
| BACE1+ | **4DJY** | **0.30** | **2.52** | **-2.41** | **0.34** |
|  | 4DJW | 0.07 | 2.34 | -2.11 | -0.23 |
|  | AlphaFold2 (BACE1+) | 0.35 | 2.17 | -2.05 | 0.41 |

**Effect of using AlphaFold2 structures**

A)	B)

C)

C)

**Supplementary Figure 2.** Correlation scatter plots of computationally predicted with experimentally determined binding affinities for A) Crystal 1, B) Crystal 2 and C) AlphaFold2 structure sets.

**Accuracy of binding free energy calculations in each protein target**

Among all the targets, c-MET achieved the highest correlation (R^2^ = 0.7) with the 5EOB structure. Its RMSE value (1.40 kcal/mol) matched that of 4R1Y and was lower than the AlphaFold2 model (2.18 kcal/mol). Similarly, MSE values for 5EOB and 4R1Y were below 1 kcal/mol (-0.62 and -0.63, respectively), while the AlphaFold2 model exhibited a higher error (-1.73 kcal/mol).

For MCL-1+, the 4WMU structure clearly outperformed both 4HW3 and AlphaFold2, yielding an R^2^ of 0.63 and RMSE/MSE values below 1 kcal/mol (0.88 and -0.36 kcal/mol respectively). In contrast, the AlphaFold2 model produced RMSE and MSE values above 2 kcal/mol (2.40 and -2.17 kcal/mol). Notably, the better results for the 4WMU crystal structure are consistent with its superior crystallographic quality (Resolution 1.55 Å for 4WMU versus 2.40 Å for 4HW3). For the rest of the targets, the observed R^2^ values were below 0.5.

For PTP1B+, both crystal structures yielded similar R^2^ values (~0.49) and RMSE values (1.02 and 0.91 kcal/mol for 2HB1 and 2QBS respectively), although 2QBS showed a smaller MSE (-0.06 versus -0.42 kcal/mol). For JNK1+, all structures gave a similar R^2^ (~0.49) but 3ELJ exhibited lower RMSE and MSE (1.31 and -1.02) than 2GMX (1.68 and -1.46 kcal/mol). Again, the superior performance of 3ELJ corresponds to its higher structural quality (1.86 Å versus 3.50 Å for 2GMX).

In the case of CDK2, the AlphaFold2 structure yielded the highest R^2^, but also higher RMSE and MSE values than the crystal structures 3DDQ and 1H1Q, both of which are medium-to-low quality according to PDB metrics. For P38+, 3FLN structure performed best across all metrics (R^2^ = 0.36, RMSE = 2.26 kcal/mol, MSE = -2.12 kcal/mol), outperforming both 3FLY and the AlphaFold2 model, which exhibited RMSE and MSE values exceeding 3 kcal/mol.

For Thrombin+, 2DA structure achieved the highest correlation (R^2^ = 0.41) while for Tyk2, 4GIH showed the highest correlation (R^2^ of 0.20), though with high RMSE (2.59 kcal/mol) and MSE (-2.30 kcal/mol). The BACE1+ target proved especially challenging due to its flexible binding pocket, variable aspartate protonation state, and ligand tautomerism [39]. The 4DJY structure provided the best results (R^2^ = 0.38, RMSE = 2.21 kcal/mol and MSE = 2.10 kcal/mol).

**Supplementary Table 10.** List of R^2^, RMSE, MSE and Kendall’s Tau values for the binding score of each crystal structure with rigid docking, GBVI scoring function, Amber10:EHT force field and TOP1 pose. The structures are grouped as ‘Crystal 1’ or ‘Crystal 2’ based on the R^2^ value, with Crystal 1 exhibiting a higher correlation with experimental binding affinities compared to Crystal 2.

|  | Target | Structure | Resolution [Å] | R^2^ | RMSE [kcal/mol] | MSE [kcal/mol] | Kendall’s Tau |
| --- | --- | --- | --- | --- | --- | --- | --- |
| Crystal 1 | c-MET | 5EOB | 1.75 | 0.70 | 1.40 | -0.63 | 0.69 |
|  | MCL-1+ | 4WMU | 1.55 | 0.63 | 0.88 | -0.36 | 0.59 |
|  | PTP1B+ | 2QBS | 2.10 | 0.49 | 0.91 | -0.06 | 0.52 |
|  | JNK1+ | 3ELJ | 1.86 | 0.49 | 1.31 | -1.02 | 0.53 |
|  | Thrombin+ | 2ZDA | 1.73 | 0.41 | 0.98 | -0.54 | 0.52 |
|  | BACE1+ | 4DJY | 1.86 | 0.38 | 2.21 | -2.10 | 0.39 |
|  | P38+ | 3FLN | 1.90 | 0.36 | 2.26 | -2.12 | 0.44 |
|  | CDK2 | 3DDQ | 1.80 | 0.22 | 1.62 | -1.22 | 0.43 |
|  | Tyk2 | 4GIH | 2.00 | 0.20 | 2.59 | -2.30 | 0.33 |
| Crystal 2 | c-MET | 4R1Y | 2.00 | 0.69 | 1.40 | -0.62 | 0.70 |
|  | MCL-1+ | 4HW3 | 2.40 | 0.45 | 0.91 | 0.01 | 0.46 |
|  | PTP1B+ | 2HB1 | 2.00 | 0.48 | 1.02 | -0.42 | 0.46 |
|  | JNK1+ | 2GMX | 3.50 | 0.48 | 1.68 | -1.46 | 0.51 |
|  | Thrombin+ | 5JZY | 1.27 | 0.10 | 0.85 | -0.07 | 0.24 |
|  | BACE1+ | 4DJW | 1.90 | 0.02 | 2.22 | -2.04 | 0.00 |
|  | P38+ | 3FLY | 1.80 | 0.30 | 2.54 | -2.40 | 0.37 |
|  | CDK2 | 1H1Q | 2.50 | 0.13 | 1.90 | -1.54 | 0.27 |
|  | Tyk2 | 3LXP | 1.65 | 0.04 | 2.56 | -2.19 | -0.10 |

**Supplementary Table 11.** List of R^2^, RMSE, MSE and Kendall’s Tau values for the binding score of each AlphaFold2 structure with rigid docking, GBVI scoring function, Amber10:EHT force field and TOP1 pose.

|  | Target | R^2^ | RMSE [kcal/mol] | MSE [kcal/mol] | Kendall’s Tau |
| --- | --- | --- | --- | --- | --- |
| AlphaFold2 | c-MET | 0.50 | 2.18 | -1.73 | 0.51 |
|  | MCL-1+ | 0.21 | 2.40 | -2.17 | 0.37 |
|  | PTP1B+ | 0.02 | 1.62 | -0.67 | 0.14 |
|  | JNK1+ | 0.47 | 1.53 | -1.28 | 0.52 |
|  | Thrombin+ | 0.00 | 0.97 | -0.38 | 0.33 |
|  | BACE1+ | 0.15 | 2.01 | -1.85 | 0.16 |
|  | P38+ | 0.21 | 3.34 | -3.22 | 0.29 |
|  | CDK2 | 0.36 | 2.05 | -1.79 | 0.40 |
|  | Tyk2 | 0.06 | 2.81 | -2.49 | -0.32 |

Ligand binding pose accuracy, evaluated by comparing the RMSD of docked ligand heavy atoms against experimental crystal poses, confirms this trend. A total of 25 ligands were assessed using rigid docking with the GBVI scoring function and Amber10:EHT force field. The results are summarized in Supplementary Table 12. AlphaFold2 models achieved RMSD values under 2.0 Å for only three ligands (CDK2 and P38+ targets), whereas the RMSDs of other ligands ranged from ~2.0 to 9.0 Å. Representative binding poses for all targets are shown in Supplementary Figure 3.

**Supplementary Table 12.** List of RMSD values between the heavy atoms of the ligands in the docked complexes to those in the experimental ligand poses across all tested structures. The “+” symbol denotes ligands which were missing in Wang et al. [14]

| Ligand | Experimental pose for the ligand considered | Crystal 1 structure ligand RMSD [Å] | Crystal 2 structure ligand RMSD [Å] | AlphaFold2 structure ligand RMSD [Å] |
| --- | --- | --- | --- | --- |
| c-MET 1 | 4R1Y | 2.1 | 0.9 | 8.2 |
| c-MET 22 | 4R1V | 0.8 | 1.5 | 9.1 |
| MCL-1+ 53 | 4HW2 | 0.3 | 0.3 | 6.3 |
| MCL-1+ 60 | 4HW3 | 0.5 | 0.2 | 6.4 |
| PTP1B+ 20669 | 2QBR | 1.5 | 6.0 | 8.8 |
| PTP1B+ 20670 | 2QBS | 0.7 | 1.3 | 4.0 |
| PTP1B+ 23330 | 2QBQ | 1.3 | 1.5 | 4.3 |
| PTP1B+ 20667 | 2QBP | 0.7 | 1.8 | 7.7 |
| JNK1+ 17124-1 | 2GMX | 6.7 | 9.0 | 6.6 |
| BACE1+ CAT-1+ | 4DJU | 5.4 | 3.9 | 6.0 |
| BACE1+ CAT-4b | 4DJV | 1.0 | 1.5 | 5.4 |
| BACE1+ CAT-4j | 4DJW | 3.6 | 0.8 | 5.2 |
| BACE1+ CAT-17h | 4DJX | 0.4 | 0.8 | 7.0 |
| BACE1+ CAT-43 | 4DJY | 0.4 | 1.1 | 7.1 |
| CDK2 1h1q | 1H1Q | 1.2 | 1.2 | 2.0 |
| CDK2 1h1r | 1H1R | 1.2 | 0.7 | 1.9 |
| CDK2 1h1s | 1H1S | 1.7 | 1.9 | 7.1 |
| P38+ p38-3lfn | 3FLN | 1.3 | 0.4 | 1.9 |
| P38+ p38-3fly | 3FLY | 0.5 | 0.4 | 0.5 |
| P38+ p38-3fmh | 3FMH | 2.5 | 2.5 | 8.9 |
| P38+ p38-3fmk | 3FMK | 2.2 | 0.7 | 8.6 |
| Thrombin+ 1b | 2ZC9 | 7.0 | 7.8 | 7.3 |
| Thrombin+ 4+ | 2ZDA | 1.2 | 0.8 | 8.1 |
| Thrombin+ 5 | 2ZFF | 7.4 | 7.7 | 7.5 |
| Tyk2 ejmc_46 | 4GIH | 0.4 | 7.1 | 8.6 |

A)

B)

C)

D)

E)

F)

G)

**Supplementary Figure 3.** Binding poses of ligands in the experimentally determined crystal structures (green-crystallized), Crystal 1 structure (orange-docked), Crystal 2 structure (purple-docked) and AlphaFold2 structure (cyan-docked) with ligand RMSD values [Å]. A) c-MET 22, B) MCL-1+ 53, C) Thrombin+ 1b, D) Tyk2 ejmc_46, E) JNK1+ 17124-1 and F) CDK2 1h1r. G) Scatter plot of RMSD versus |ΔG_pred_ − ΔG_exp_|.

**Effect of induced fit docking**

Among all systems, c-MET (5EOB) showed the highest R^2^ (0.70). Although 4R1Y structure had slightly lower R² (0.59), its RMSE and MSE values were comparable (1.33 versus 1.35 kcal/mol and -0.32 versus -0.47 kcal/mol). For Thrombin+, 5JZY outperformed both 2ZDA and AlphaFold2 (R^2^ = 0.65 versus 0.06 and 0.04). CDK2, (3DDQ) achieved R^2^ = 0.57 with RMSE below 1 kcal/mol. In JNK1+, both crystal structures performed equally (R^2^ = 0.54), while AlphaFold2 yielded a slightly lower R^2^ value (0.48). PTP1B+ results were comparable across models. For MCL-1+, 4HW3 produced the best correlation (0.39). In P38+, 3FLY and 3FLN performed similarly (R^2^ ~ 0.33), with AlphaFold2 slightly lower (0.28). Tyk2 and BACE1+ exhibited low correlations and high RMSE/MSE values for all structures.

A)	B)

C)	D)

**Supplementary Figure 4.** Bar charts of R^2^, RMSE, MSE and Kendall’s Tau values for the binding score of each structure with induced fit docking, GBVI WSA dG scoring function, Amber10:EHT force field and TOP1 pose on the JACS Schrödinger+. The structures are grouped as ‘Crystal 1’ or ‘Crystal 2’ based on the R^2^ value, with Crystal 1 exhibiting a higher correlation with experimental binding affinities compared to Crystal 2. A) R^2^, B) RMSE, C) MSE and D) Kendall’s Tau.

**Comparison of binding free energy calculations using rigid docking between the JACS Schrödinger and the JACS Schrödinger+ datasets**

To ensure a consistent comparison with methods previously assessed using the JACS Schrödinger dataset from Wang et al., metrics for each target, with the ligand dataset from JACS Schrödinger dataset (BACE1, Thrombin, JNK1, MCL-1, PTP1B, and P38), are presented (Supplementary Table 13) [14]. For MCL-1, both crystal structures yielded similar results in both datasets, while the R² of AlphaFold2 declined from 0.21 to 0.10. In PTP1B, the R² of 2QBS improved from 0.49 to 0.60, whereas the results for 2HB1 and AlphaFold2 remained consistent. For JNK1, 3ELJ maintained its performance, but 2GMX and AlphaFold2 displayed decreased R² values (0.48 to 0.34 and 0.47 to 0.30, respectively). With respect to Thrombin, the RMSE of 2ZDA decreased (0.98 to 0.57 kcal/mol), while for 5JZY the R² increased (0.10 to 0.30). The AlphaFold2 structure of Thrombin showed substantial improvements across all metrics, as R² increased from 0.00 to 0.53, RMSE decreased from 0.97 to 0.43 kcal/mol and MSE improved from -0.38 to -0.13 kcal/mol. Furthermore, no differences were found for BACE1 and P38 targets. These results suggest that only a limited number of structures exhibited notable differences between the two datasets.

**Supplementary Table 13.** List of R^2^, RMSE, MSE and Kendall’s Tau values for the JACS Schrödinger dataset from Wang et al., using the binding score of each structure with rigid docking, GBVI WSA dG scoring function, Amber10:EHT force field and TOP1 pose [14]. The structures are grouped as ‘Crystal 1’ or ‘Crystal 2’ based on the R^2^ value, with Crystal 1 exhibiting a higher correlation with experimental binding affinities compared to Crystal 2.

|  | Target | Structure | R^2^ | RMSE [kcal/mol] | MSE [kcal/mol] | Kendall’s Tau |
| --- | --- | --- | --- | --- | --- | --- |
| Crystal 1 | MCL-1 | 4WMU | 0.57 | 0.90 | -0.42 | 0.56 |
|  | PTP1B | 2QBS | 0.60 | 0.88 | -0.14 | 0.57 |
|  | JNK1 | 3ELJ | 0.43 | 1.48 | -1.33 | 0.53 |
|  | Thrombin | 2ZDA | 0.35 | 0.57 | -0.33 | 0.45 |
|  | BACE1 | 4DJY | 0.39 | 2.20 | -2.11 | 0.37 |
|  | P38 | 3FLN | 0.36 | 2.26 | -2.11 | 0.44 |
| Crystal 2 | MCL-1 | 4HW3 | 0.41 | 0.89 | -0.06 | 0.42 |
|  | PTP1B | 2HB1 | 0.54 | 1.06 | -0.50 | 0.46 |
|  | JNK1 | 2GMX | 0.34 | 1.91 | -1.79 | 0.48 |
|  | Thrombin | 5JZY | 0.30 | 0.46 | 0.15 | 0.24 |
|  | BACE1 | 4DJW | 0.00 | 2.21 | -2.03 | -0.02 |
|  | P38 | 3FLY | 0.30 | 2.53 | -2.39 | 0.38 |
| AlphaFold2 | MCL-1 | - | 0.10 | 2.44 | -2.22 | 0.27 |
|  | PTP1B | - | 0.02 | 1.71 | -0.79 | 0.12 |
|  | JNK1 | - | 0.30 | 1.55 | -1.37 | 0.46 |
|  | Thrombin | - | 0.53 | 0.43 | -0.13 | 0.60 |
|  | BACE1 | - | 0.11 | 2.01 | -1.84 | 0.15 |
|  | P38 | - | 0.20 | 3.33 | -3.21 | 0.29 |

**Comparison of binding free energy calculations using induced fit docking between the JACS Schrödinger and the JACS Schrödinger+ datasets**

Metrics for each target using induced fit docking on the JACS Schrödinger dataset (BACE1, Thrombin, JNK1, MCL-1, CDK2, PTP1B, P38, and Tyk2), are presented in Supplementary Figure 5. For MCL-1, both crystal structures exhibited a reduction in R², while the AlphaFold2 model produced consistent results across the datasets. In contrast, for PTP1B, the R² of the 2QBS structure improved to 0.55, and 2HB1 and AlphaFold2 increased to 0.46 and 0.53 respectively.

For JNK1, 3ELJ performed better than 2GMX with the JACS Schrödinger+ dataset, whereas the reverse trend was observed in the JACS Schrödinger dataset. Interestingly, RMSE and MSE values remained largely unchanged across datasets for each crystal structure. In the case of Thrombin, performance decreased substantially for the 5JZY structure (from R^2^ = 0.65 to 0.21 and RMSE from 0.42 to 0.74 kcal/mol). For both BACE1 and P38, no notable differences were observed between datasets.

A) B)

C)	D)

**Supplementary Figure 5.** List of metrics for the binding score of each structure with induced fit docking, GBVI WSA dG scoring function, Amber10:EHT force field and TOP1 pose on the JACS Schrödinger dataset from Wang et al [14]. The structures are grouped as ‘Crystal 1’ or ‘Crystal 2’ based on the R^2^ value, with Crystal 1 exhibiting a higher correlation with experimental binding affinities compared to Crystal 2. A) R^2^, B) RMSE, C) MSE and D) Kendall’s Tau.

**Influence of water molecules presence in the active site**

To assess the influence of active site water molecules on the accuracy of binding score predictions for the five protein targets (CDK2, c-MET, JNK1+, P38+, and Thrombin+), analyses were performed on the corresponding Crystal 1 structures in the absence of water. Binding scores were calculated using the Amber10:EHT force field, the GBVI scoring function, and the lowest energy binding pose. The resulting metrics (R_mean_^2^, RMSE_mean_, MSE_mean_, and Kendall’s Tau_mean_) are summarized in Supplementary Table 14.

The R_mean_² values obtained from docking without water molecules were nearly identical to those from water-containing structures, while RMSE_mean_ and MSE_mean_ values were likewise comparable.

**Supplementary Table 14.** Comparison of binding scores between models including active site water molecules and those without, for the set of protein targets (CDK2, c-MET, JNK1+, P38+ and Thrombin+) using rigid docking, Amber10:EHT force field, GBVI scoring function and the lowest binding energy pose.

| Water molecules | R_mean_^2^ $\pm$ Standard deviation | RMSE_mean_ [kcal / mol] $\pm$ Standard deviation | MSE_mean_ [kcal / mol] $\pm$ Standard deviation | Kendall’s Tau_mean_ $\pm$ Standard deviation |
| --- | --- | --- | --- | --- |
| Presence | 0.44 $\pm$ 0.16 | 1.51 $\pm$ 0.43 | -1.11 $\pm$ 0.56 | 0.52 $\pm$ 0.09 |
| Absence | 0.45 $\pm$ 0.15 | 1.51 $\pm$ 0.48 | -1.11 $\pm$ 0.61 | 0.53 $\pm$ 0.10 |

Furthermore, the performance of docking in a structure with a misplaced water molecule was evaluated using the 2ZDA structure of Thrombin+ (Supplementary Table 15). The position of the water was adjusted from the S1 pocket closer to the S3 pocket of Thrombin, to the center of the active site, using a restrained energy minimization, with a weight of 100, in the constrained receptor. The R² value obtained from docking in the structure with the misplaced water molecule was lower compared to those from the structures that either contained the water in the S1 pocket, or the water was absent. On the contrary, the RMSE and MSE values were higher in the structure with the misplaced water relative to the structures that either contained the water in the S1 pocket, or the water was absent.

**Supplementary Table 15.** Comparison of binding scores from three 2ZDA models of Thrombin+ with an active site water molecule on the S1 pocket, or between S1 and S3 pockets or the water molecule absent, using rigid docking, Amber10:EHT force field, GBVI scoring function and the lowest binding energy pose.

| Water molecule in S1 pocket of 2ZDA | R^2^ | RMSE [kcal / mol] | MSE [kcal / mol] | Kendall’s Tau |
| --- | --- | --- | --- | --- |
| Present | 0.41 | 0.98 | -0.54 | 0.52 |
| Absent | 0.41 | 0.90 | -0.46 | 0.55 |
| Misplaced | 0.20 | 1.23 | -0.91 | 0.45 |

**Supplementary Table 16.** List of ligands added to the JACS Schrödinger dataset from Wang et al. [14].

| Target | Ligand | Binding affinity [kcal / mol] |
| --- | --- | --- |
| MCL-1 [19] | mcl1_friberg_21 | -6.69 |
|  | mcl1_friberg_22 | -6.85 |
|  | mcl1_friberg_24 | -6.85 |
|  | mcl1_friberg_25 | -6.60 |
|  | mcl1_friberg_55 | -9.80 |
| PTP1B [20] | ptp1b_wilson_09 | -8.15 |
|  | ptp1b_wilson_26 | -7.37 |
|  | ptp1b_wilson_29 | -8.86 |
| Thrombin [21] | thrombin_baum_14_4_2zda | -11.02 |
| BACE1 [22] | bace1_merck_01_1_4dju | -7.43 |
|  | bace1_merck_06_4e | -8.38 |
|  | bace1_merck_07_4f | -8.90 |
|  | bace1_merck_08_4g | -8.54 |
|  | bace1_merck_09_4h | -8.77 |
|  | bace1_merck_31_13l | -6.82 |
|  | bace1_merck_44_19 | -10.47 |
| P38 [23] | p38_goldstein_29_2cc_3fls | -11.32 |
| JNK1 [24] | AZ1 | -8.72 |
|  | AZ2 | -10.63 |
|  | AZ3 | -9.71 |
|  | jnk1_abbot_00_6a | -8.32 |
|  | jnk1_abbot_01_6b | -8.77 |
|  | jnk1_abbot_02_6c | -7.34 |
|  | jnk1_abbot_03_6d | -7.80 |
|  | jnk1_abbot_21 | -6.79 |
|  | jnk1_abbot_22 | -6.79 |
|  | jnk1_abbot_23 | -6.79 |
|  | jnk1_abbot_24 | -7.57 |
|  | jnk1_abbot_25 | -7.41 |
|  | jnk1_abbot_26 | -7.07 |
|  | jnk1_abbot_27 | -9.24 |
|  | jnk1_abbot_28 | -8.55 |
|  | jnk1_abbot_29 | -8.93 |
|  | jnk1_abbot_30 | -8.87 |
|  | jnk1_abbot_31 | -9.73 |
|  | jnk1_abbot_32 | -9.59 |
|  | jnk1_abbot_33 | -7.84 |
|  | jnk1_abbot_34 | -8.84 |
|  | jnk1_abbot_36_19a | -6.79 |
|  | jnk1_abbot_37_19b | -7.23 |
|  | jnk1_abbot_38_19c | -8.22 |
|  | jnk1_abbot_39_19d | -7.72 |
|  | jnk1_abbot_40_19e | -7.69 |
|  | jnk1_abbot_44 | -6.86 |
|  | jnk1_abbot_45 | -7.64 |
|  | jnk1_abbot_46 | -8.21 |
|  | jnk1_abbot_47 | -7.77 |
|  | jnk1_abbot_48 | -6.79 |
|  | jnk1_abbot_49 | -7.59 |
|  | jnk1_abbot_50 | -6.79 |
|  | jnk1_abbot_51 | -6.79 |
|  | jnk1_abbot_52 | -6.79 |
|  | jnk1_abbot_53 | -6.79 |
|  | jnk1_abbot_54 | -6.79 |
|  | jnk1_abbot_55 | -10.12 |

**Supplementary Table 17. Mean performance across all targets (Crystal 1, MOE ligands).**

| **Method** | **R²** | **RMSE (kcal/mol)** | **Mean signed error (kcal/mol)** | **Kendall's τ** |
| --- | --- | --- | --- | --- |
| MOE rigid | 0.39 ± 0.14 | 1.56 ± 0.74 | -1.25 ± 0.87 | 0.46 ± 0.09 |
| MW | 0.23 ± 0.18 | 0.85 ± 0.23 | 0.00 ± 0.00 | 0.34 ± 0.16 |
| Smina | 0.22 ± 0.15 | 2.35 ± 1.06 | 2.02 ± 1.26 | 0.12 ± 0.32 |
| AutoDock Vina | 0.10 ± 0.08 | 1.46 ± 0.45 | 0.61 ± 0.99 | 0.11 ± 0.18 |
| GNINA | 0.19 ± 0.17 | 1.54 ± 0.44 | 0.28 ± 1.18 | 0.25 ± 0.20 |
| DOCK6 | 0.49 ± 0.16 | — | — | 0.48 ± 0.10 |

**Supplementary Table 18. Mean performance across all targets (Crystal 1, Schrödinger ligands).**

| **Method** | **R²** | **RMSE (kcal/mol)** | **Mean signed error (kcal/mol)** | **Kendall's τ** |
| --- | --- | --- | --- | --- |
| MW | 0.23 ± 0.18 | 0.85 ± 0.23 | 0.00 ± 0.00 | 0.34 ± 0.16 |
| Smina | 0.14 ± 0.14 | 2.33 ± 1.02 | 1.96 ± 1.24 | 0.14 ± 0.27 |
| AutoDock Vina | 0.09 ± 0.07 | 1.48 ± 0.46 | 0.57 ± 1.03 | 0.10 ± 0.24 |
| GNINA | 0.17 ± 0.14 | 1.66 ± 0.47 | 0.22 ± 1.19 | 0.12 ± 0.27 |
| DOCK6 | 0.49 ± 0.17 | — | — | 0.50 ± 0.11 |

**Supplementary Table 19. Mean performance across all targets (Crystal 2, Schrödinger ligands).**

| **Method** | **R²** | **RMSE (kcal/mol)** | **Mean signed error (kcal/mol)** | **Kendall's τ** |
| --- | --- | --- | --- | --- |
| MW | 0.23 ± 0.18 | 0.85 ± 0.23 | 0.00 ± 0.00 | 0.34 ± 0.16 |
| Smina | 0.21 ± 0.19 | 2.58 ± 1.13 | 2.19 ± 1.44 | 0.11 ± 0.31 |
| AutoDock Vina | 0.19 ± 0.17 | 1.55 ± 0.45 | 0.63 ± 1.10 | 0.10 ± 0.32 |
| GNINA | 0.22 ± 0.22 | 1.63 ± 0.46 | 0.38 ± 1.30 | 0.26 ± 0.28 |
| DOCK6 | 0.18 ± 0.20 | — | — | 0.19 ± 0.27 |

**Supplementary Table 20. Per-target R² values (Figure 7).**

| **Target** | **Crystal 1 PDB** | **N** | **MOE rigid** | **MW** | **Smina** | **AutoDock Vina** | **GNINA** | **DOCK6** |
| --- | --- | --- | --- | --- | --- | --- | --- | --- |
| BACE1+ | 4DJY | 36 | 0.39 | 0.02 | 0.15 | 0.00 | 0.05 | 0.35 |
| CDK2 | 3DDQ | 16 | 0.22 | 0.24 | 0.41 | 0.17 | 0.17 | 0.54 |
| JNK1 | 3ELJ | 21 | 0.43 | 0.16 | 0.20 | 0.08 | 0.01 | 0.54 |
| MCL-1 | 4WMU | 42 | 0.57 | 0.29 | 0.02 | 0.04 | 0.05 | 0.53 |
| P38 | 3FLN | 34 | 0.36 | 0.32 | 0.34 | 0.18 | 0.39 | 0.30 |
| PTP1B | 2QBS | 23 | 0.60 | 0.55 | 0.38 | 0.11 | 0.27 | 0.47 |
| Thrombin+ | 2ZDA | 11 | 0.35 | 0.22 | 0.21 | 0.20 | 0.48 | 0.80 |
| Tyk2 | 4GIH | 16 | 0.20 | 0.00 | 0.03 | 0.01 | 0.09 | 0.36 |
| **Mean ± SD** |  |  | **0.39 ± 0.14** | **0.23 ± 0.18** | **0.22 ± 0.15** | **0.10 ± 0.08** | **0.19 ± 0.17** | **0.49 ± 0.16** |

**Supplementary Table 21. Per-target RMSE values (Figure 7).**

| **Target** | **Crystal 1 PDB** | **N** | **MOE rigid** | **MW** | **Smina** | **AutoDock Vina** | **GNINA** | **DOCK6** |
| --- | --- | --- | --- | --- | --- | --- | --- | --- |
| BACE1+ | 4DJY | 36 | 2.20 | 0.76 | 1.58 | 1.22 | 1.50 | — |
| CDK2 | 3DDQ | 16 | 1.62 | 1.03 | 2.76 | 1.67 | 1.46 | — |
| JNK1 | 3ELJ | 21 | 1.48 | 0.77 | 2.03 | 1.33 | 2.26 | — |
| MCL-1 | 4WMU | 42 | 0.90 | 0.88 | 1.15 | 1.33 | 1.39 | — |
| P38 | 3FLN | 34 | 2.26 | 0.82 | 2.19 | 1.58 | 0.80 | — |
| PTP1B | 2QBS | 23 | 0.88 | 0.86 | 4.25 | 1.41 | 1.99 | — |
| Thrombin+ | 2ZDA | 11 | 0.57 | 0.46 | 1.46 | 0.79 | 1.62 | — |
| Tyk2 | 4GIH | 16 | 2.59 | 1.26 | 3.39 | 2.36 | 1.28 | — |
| **Mean ± SD** |  |  | **1.56 ± 0.74** | **0.85 ± 0.23** | **2.35 ± 1.06** | **1.46 ± 0.45** | **1.54 ± 0.44** | **—** |

**Supplementary Table 22. Per-target mean signed error values (Figure 7).**

| **Target** | **Crystal 1 PDB** | **N** | **MOE rigid** | **MW** | **Smina** | **AutoDock Vina** | **GNINA** | **DOCK6** |
| --- | --- | --- | --- | --- | --- | --- | --- | --- |
| BACE1+ | 4DJY | 36 | -2.11 | 0.00 | 1.16 | -0.85 | 0.87 | — |
| CDK2 | 3DDQ | 16 | -1.22 | -0.00 | 2.59 | 1.26 | -0.95 | — |
| JNK1 | 3ELJ | 21 | -1.33 | 0.00 | 1.74 | 0.93 | 1.90 | — |
| MCL-1 | 4WMU | 42 | -0.42 | -0.00 | 0.02 | -0.76 | 0.23 | — |
| P38 | 3FLN | 34 | -2.11 | 0.00 | 2.03 | 1.29 | 0.06 | — |
| PTP1B | 2QBS | 23 | -0.14 | 0.00 | 4.12 | 0.71 | 1.65 | — |
| Thrombin+ | 2ZDA | 11 | -0.33 | -0.00 | 1.37 | 0.36 | -1.55 | — |
| Tyk2 | 4GIH | 16 | -2.30 | -0.00 | 3.08 | 1.95 | 0.05 | — |
| **Mean ± SD** |  |  | **-1.25 ± 0.87** | **0.00 ± 0.00** | **2.02 ± 1.26** | **0.61 ± 0.99** | **0.28 ± 1.18** | **—** |

**Supplementary Table 23. Per-target Kendall's τ values (Figure 7).**

| **Target** | **Crystal 1 PDB** | **N** | **MOE rigid** | **MW** | **Smina** | **AutoDock Vina** | **GNINA** | **DOCK6** |
| --- | --- | --- | --- | --- | --- | --- | --- | --- |
| BACE1+ | 4DJY | 36 | 0.37 | 0.17 | -0.28 | -0.05 | -0.01 | 0.35 |
| CDK2 | 3DDQ | 16 | 0.43 | 0.36 | 0.53 | 0.15 | 0.32 | 0.53 |
| JNK1 | 3ELJ | 21 | 0.53 | 0.37 | -0.28 | -0.19 | -0.03 | 0.59 |
| MCL-1 | 4WMU | 42 | 0.56 | 0.36 | 0.07 | 0.14 | 0.15 | 0.52 |
| P38 | 3FLN | 34 | 0.44 | 0.39 | 0.40 | 0.32 | 0.43 | 0.37 |
| PTP1B | 2QBS | 23 | 0.57 | 0.51 | 0.31 | 0.23 | 0.42 | 0.46 |
| Thrombin+ | 2ZDA | 11 | 0.45 | 0.51 | 0.34 | 0.27 | 0.45 | 0.64 |
| Tyk2 | 4GIH | 16 | 0.33 | 0.03 | -0.11 | -0.03 | 0.25 | 0.40 |
| **Mean ± SD** |  |  | **0.46 ± 0.09** | **0.34 ± 0.16** | **0.12 ± 0.32** | **0.11 ± 0.18** | **0.25 ± 0.20** | **0.48 ± 0.10** |

**Supplementary Table 24. Per-target R² values (Crystal 1, Schrödinger ligands).**

| **Target** | **Crystal 1 PDB** | **N** | **MW** | **Smina** | **AutoDock Vina** | **GNINA** | **DOCK6** |
| --- | --- | --- | --- | --- | --- | --- | --- |
| BACE1+ | 4DJY | 36 | 0.02 | 0.12 | 0.06 | 0.02 | 0.32 |
| CDK2 | 3DDQ | 16 | 0.24 | 0.20 | 0.17 | 0.36 | 0.59 |
| JNK1 | 3ELJ | 21 | 0.16 | 0.08 | 0.07 | 0.22 | 0.40 |
| MCL-1 | 4WMU | 42 | 0.29 | 0.03 | 0.03 | 0.06 | 0.50 |
| P38 | 3FLN | 34 | 0.32 | 0.32 | 0.18 | 0.38 | 0.31 |
| PTP1B | 2QBS | 23 | 0.55 | 0.36 | 0.06 | 0.02 | 0.50 |
| Thrombin+ | 2ZDA | 11 | 0.22 | 0.02 | 0.18 | 0.18 | 0.83 |
| Tyk2 | 4GIH | 16 | 0.00 | 0.00 | 0.01 | 0.10 | 0.45 |
| **Mean ± SD** |  |  | **0.23 ± 0.18** | **0.14 ± 0.14** | **0.09 ± 0.07** | **0.17 ± 0.14** | **0.49 ± 0.17** |

**Supplementary Table 25. Per-target RMSE values (Crystal 1, Schrödinger ligands).**

| **Target** | **Crystal 1 PDB** | **N** | **MW** | **Smina** | **AutoDock Vina** | **GNINA** | **DOCK6** |
| --- | --- | --- | --- | --- | --- | --- | --- |
| BACE1+ | 4DJY | 36 | 0.76 | 1.51 | 1.43 | 1.57 | — |
| CDK2 | 3DDQ | 16 | 1.03 | 2.79 | 1.67 | 1.42 | — |
| JNK1 | 3ELJ | 21 | 0.77 | 1.89 | 1.32 | 2.20 | — |
| MCL-1 | 4WMU | 42 | 0.88 | 1.13 | 1.35 | 1.46 | — |
| P38 | 3FLN | 34 | 0.82 | 2.20 | 1.58 | 0.80 | — |
| PTP1B | 2QBS | 23 | 0.86 | 4.17 | 1.42 | 2.24 | — |
| Thrombin+ | 2ZDA | 11 | 0.46 | 1.65 | 0.70 | 1.89 | — |
| Tyk2 | 4GIH | 16 | 1.26 | 3.27 | 2.36 | 1.67 | — |
| **Mean ± SD** |  |  | **0.85 ± 0.23** | **2.33 ± 1.02** | **1.48 ± 0.46** | **1.66 ± 0.47** | **—** |

**Supplementary Table 26. Per-target mean signed error values (Crystal 1, Schrödinger ligands).**

| **Target** | **Crystal 1 PDB** | **N** | **MW** | **Smina** | **AutoDock Vina** | **GNINA** | **DOCK6** |
| --- | --- | --- | --- | --- | --- | --- | --- |
| BACE1+ | 4DJY | 36 | 0.00 | 0.96 | -1.04 | 1.00 | — |
| CDK2 | 3DDQ | 16 | -0.00 | 2.57 | 1.26 | -0.94 | — |
| JNK1 | 3ELJ | 21 | 0.00 | 1.61 | 0.92 | 1.35 | — |
| MCL-1 | 4WMU | 42 | -0.00 | 0.05 | -0.77 | 0.16 | — |
| P38 | 3FLN | 34 | 0.00 | 2.04 | 1.29 | 0.05 | — |
| PTP1B | 2QBS | 23 | 0.00 | 4.03 | 0.61 | 1.82 | — |
| Thrombin+ | 2ZDA | 11 | -0.00 | 1.46 | 0.36 | -1.79 | — |
| Tyk2 | 4GIH | 16 | -0.00 | 2.98 | 1.95 | 0.13 | — |
| **Mean ± SD** |  |  | **0.00 ± 0.00** | **1.96 ± 1.24** | **0.57 ± 1.03** | **0.22 ± 1.19** | **—** |

**Supplementary Table 27. Per-target Kendall's τ values (Crystal 1, Schrödinger ligands).**

| **Target** | **Crystal 1 PDB** | **N** | **MW** | **Smina** | **AutoDock Vina** | **GNINA** | **DOCK6** |
| --- | --- | --- | --- | --- | --- | --- | --- |
| BACE1+ | 4DJY | 36 | 0.17 | -0.26 | -0.20 | -0.09 | 0.35 |
| CDK2 | 3DDQ | 16 | 0.36 | 0.41 | 0.15 | 0.43 | 0.60 |
| JNK1 | 3ELJ | 21 | 0.37 | -0.20 | -0.19 | -0.18 | 0.55 |
| MCL-1 | 4WMU | 42 | 0.36 | 0.12 | 0.12 | 0.22 | 0.51 |
| P38 | 3FLN | 34 | 0.39 | 0.39 | 0.32 | 0.47 | 0.37 |
| PTP1B | 2QBS | 23 | 0.51 | 0.42 | 0.13 | 0.16 | 0.50 |
| Thrombin+ | 2ZDA | 11 | 0.51 | 0.25 | 0.49 | 0.20 | 0.67 |
| Tyk2 | 4GIH | 16 | 0.03 | 0.03 | -0.03 | -0.28 | 0.42 |
| **Mean ± SD** |  |  | **0.34 ± 0.16** | **0.14 ± 0.27** | **0.10 ± 0.24** | **0.12 ± 0.27** | **0.50 ± 0.11** |

**Supplementary Table 28. Per-target R² values (Crystal 2, Schrödinger ligands).**

| **Target** | **Crystal 2 PDB** | **N** | **MW** | **Smina** | **AutoDock Vina** | **GNINA** | **DOCK6** |
| --- | --- | --- | --- | --- | --- | --- | --- |
| BACE1+ | 4DJW | 36 | 0.02 | 0.15 | 0.09 | 0.02 | 0.02 |
| CDK2 | 1H1Q | 16 | 0.24 | 0.03 | 0.05 | 0.35 | 0.01 |
| JNK1 | 2GMX | 21 | 0.16 | 0.01 | 0.26 | 0.22 | 0.27 |
| MCL-1 | 4HW3 | 42 | 0.29 | 0.01 | 0.02 | 0.08 | 0.18 |
| P38 | 3FLY | 34 | 0.32 | 0.52 | 0.34 | 0.24 | 0.28 |
| PTP1B | 2HB1 | 23 | 0.55 | 0.32 | 0.26 | 0.11 | 0.59 |
| Thrombin+ | 5JZY | 11 | 0.22 | 0.37 | 0.49 | 0.68 | 0.07 |
| Tyk2 | 3LXP | 16 | 0.00 | 0.25 | 0.03 | 0.03 | 0.00 |
| **Mean ± SD** |  |  | **0.23 ± 0.18** | **0.21 ± 0.19** | **0.19 ± 0.17** | **0.22 ± 0.22** | **0.18 ± 0.20** |

**Supplementary Table 29. Per-target RMSE values (Crystal 2, Schrödinger ligands).**

| **Target** | **Crystal 2 PDB** | **N** | **MW** | **Smina** | **AutoDock Vina** | **GNINA** | **DOCK6** |
| --- | --- | --- | --- | --- | --- | --- | --- |
| BACE1+ | 4DJW | 36 | 0.76 | 2.61 | 1.17 | 1.77 | — |
| CDK2 | 1H1Q | 16 | 1.03 | 3.14 | 1.58 | 1.25 | — |
| JNK1 | 2GMX | 21 | 0.77 | 2.68 | 1.96 | 1.32 | — |
| MCL-1 | 4HW3 | 42 | 0.88 | 1.36 | 1.75 | 1.41 | — |
| P38 | 3FLY | 34 | 0.82 | 2.25 | 1.71 | 0.97 | — |
| PTP1B | 2HB1 | 23 | 0.86 | 4.55 | 1.70 | 2.21 | — |
| Thrombin+ | 5JZY | 11 | 0.46 | 0.91 | 0.60 | 2.19 | — |
| Tyk2 | 3LXP | 16 | 1.26 | 3.13 | 1.91 | 1.90 | — |
| **Mean ± SD** |  |  | **0.85 ± 0.23** | **2.58 ± 1.13** | **1.55 ± 0.45** | **1.63 ± 0.46** | **—** |

**Supplementary Table 30. Per-target mean signed error values (Crystal 2, Schrödinger ligands).**

| **Target** | **Crystal 2 PDB** | **N** | **MW** | **Smina** | **AutoDock Vina** | **GNINA** | **DOCK6** |
| --- | --- | --- | --- | --- | --- | --- | --- |
| BACE1+ | 4DJW | 36 | 0.00 | 2.38 | -0.08 | 1.35 | — |
| CDK2 | 1H1Q | 16 | -0.00 | 2.91 | 1.08 | -0.80 | — |
| JNK1 | 2GMX | 21 | 0.00 | 2.52 | 1.62 | 1.00 | — |
| MCL-1 | 4HW3 | 42 | -0.00 | -0.38 | -1.33 | 0.26 | — |
| P38 | 3FLY | 34 | 0.00 | 2.13 | 1.51 | 0.43 | — |
| PTP1B | 2HB1 | 23 | 0.00 | 4.41 | 1.29 | 1.83 | — |
| Thrombin+ | 5JZY | 11 | -0.00 | 0.81 | -0.45 | -2.16 | — |
| Tyk2 | 3LXP | 16 | -0.00 | 2.77 | 1.36 | 1.15 | — |
| **Mean ± SD** |  |  | **0.00 ± 0.00** | **2.19 ± 1.44** | **0.63 ± 1.10** | **0.38 ± 1.30** | **—** |

**Supplementary Table 31. Per-target Kendall's τ values (Crystal 2, Schrödinger ligands).**

| **Target** | **Crystal 2 PDB** | **N** | **MW** | **Smina** | **AutoDock Vina** | **GNINA** | **DOCK6** |
| --- | --- | --- | --- | --- | --- | --- | --- |
| BACE1+ | 4DJW | 36 | 0.17 | -0.26 | -0.21 | -0.13 | 0.09 |
| CDK2 | 1H1Q | 16 | 0.36 | 0.11 | 0.20 | 0.52 | -0.12 |
| JNK1 | 2GMX | 21 | 0.37 | 0.01 | -0.45 | 0.25 | 0.42 |
| MCL-1 | 4HW3 | 42 | 0.36 | -0.06 | 0.09 | 0.18 | 0.30 |
| P38 | 3FLY | 34 | 0.39 | 0.55 | 0.38 | 0.41 | 0.40 |
| PTP1B | 2HB1 | 23 | 0.51 | 0.37 | 0.38 | 0.18 | 0.60 |
| Thrombin+ | 5JZY | 11 | 0.51 | 0.40 | 0.45 | 0.71 | -0.05 |
| Tyk2 | 3LXP | 16 | 0.03 | -0.26 | 0.00 | -0.07 | -0.10 |
| **Mean ± SD** |  |  | **0.34 ± 0.16** | **0.11 ± 0.31** | **0.10 ± 0.32** | **0.26 ± 0.28** | **0.19 ± 0.27** |
